# A Review of Fatty Acid Biosynthesis Enzyme Inhibitors as Promising Antimicrobial Drugs

**DOI:** 10.3390/ph16030425

**Published:** 2023-03-10

**Authors:** Laurie Bibens, Jean-Paul Becker, Alexandra Dassonville-Klimpt, Pascal Sonnet

**Affiliations:** Agents Infectieux, Résistance et Chimiothérapie, UR 4294, Université de Picardie-Jules-Verne, UFR de Pharmacie, 1 Rue des Louvels, CEDEX 1, F-80037 Amiens, France

**Keywords:** antimicrobials, antimicrobial resistance, fatty acid synthase system, FAS-II inhibitors

## Abstract

Resistance to antimicrobial drugs is currently a serious threat to human health. Consequently, we are facing an urgent need for new antimicrobial drugs acting with original modes of action. The ubiquitous and widely conserved microbial fatty acid biosynthesis pathway, called FAS-II system, represents a potential target to tackle antimicrobial resistance. This pathway has been extensively studied, and eleven proteins have been described. FabI (or InhA, its homologue in mycobacteria) was considered as a prime target by many teams and is currently the only enzyme with commercial inhibitor drugs: triclosan and isoniazid. Furthermore, afabicin and CG400549, two promising compounds which also target FabI, are in clinical assays to treat *Staphylococcus aureus*. However, most of the other enzymes are still underexploited targets. This review, after presenting the FAS-II system and its enzymes in *Escherichia coli*, highlights the reported inhibitors of the system. Their biological activities, main interactions formed with their targets and structure–activity relationships are presented as far as possible.

## 1. Introduction

Infectious diseases are among the main causes of death worldwide. Six of the top ten causes of death in low-income countries are still communicable diseases, including malaria, tuberculosis and HIV/AIDS [1]. Antimicrobial-resistant infections represent one the biggest public health issues [2]. Multidrug resistance is particularly worrying in Gram-negative bacteria isolated from nosocomial infections, *Escherichia coli* and *Pseudomonas aeruginosa*, for instance. In 2019, bacterial infections were associated with 7.7 million deaths, of which almost 1.27 million were directly attributable to drug resistance [2,3]. Hence, thirty-three bacteria are thought to be responsible for 13.6% of deaths. Bacterial infections are the second leading cause of death in the world, after ischemic heart disease [2]. Half of all global bacterial deaths in 2019 were due to five bacterial pathogens: *Staphylococcus aureus*, *E. coli*, *Streptococcus pneumoniae*, *Klebsiella pneumoniae* and *P. aeruginosa* [2]. In the absence of new treatments by 2050, according to the Review on Antimicrobial (ATM) Resistance, bacterial infections could lead to the deaths of 10 million people each year [4]. Parasites also raise concern and particularly *Plasmodium* spp. Among them, *Plasmodium falciparum*, the most virulent *Plasmodium* sp., was responsible for most of the 619,000 deaths reported in 2021 globally [5]. Furthermore, the decreasing effectiveness of antimalarial treatments is worrying, particularly because of the multiplication of multidrug-resistant *P. falciparum* strains. Consequently, it is urgent to develop new ATM drugs with original and selective modes of action.

To avoid cross-resistance, new drugs should be directed towards unexploited targets or vital metabolisms, e.g., adenosine triphosphate and fatty acid biosynthesis. Herein, only fatty acid biosynthesis will be explored. Fatty acids are the main constituents of bacterial and plasmodial membranes and metabolic intermediates. Their biosynthesis involves fatty acid synthase systems which are divided into two distinct molecular forms called types I and II (FAS-I and FAS-II, respectively). FAS-I is constituted of a unique multifunctional protein, whereas in FAS-II, several discrete enzymes co-exist, and each one catalyses a sole reaction. Only FAS-I is present in humans, while FAS-II is found in bacteria, mycobacteria and *P. falciparum*. FAS-II enzymes are attractive targets for ATM drug development because (i) fatty acids are essential to maintain the vital integrity of bacterial membrane, (ii) FAS-II is essential in the late liver stage development of *P. falciparum* [6], (iii) the amino acid sequences of the active sites of FAS-II enzymes are well conserved in microbial pathogens, allowing broad-spectrum activity, (iv) FAS-II does not exist in humans, limiting side effects, and (v) the crystal structures of FAS-II enzymes are available in the Protein Data Bank (PDB), allowing rational design of inhibitors. Furthermore, FAS-II enzymes are validated targets, since two commercial drugs inhibit them: **triclosan** and isoniazid [7,8].

After description of structure and functioning of the FAS-II enzymes, this review reports the biological activities, structure–activity relationships (SAR) of known FAS-II inhibitors and their main target interactions.

## 2. FAS-II Enzymes and Their Corresponding Inhibitors

**Figure 1:**
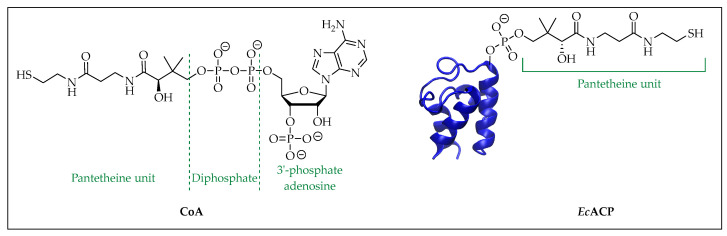
Structures of CoA and *Ec*ACP (PDB ID 6N3P) [10].

In the FAS-II system, coenzyme A (CoA, Figure 1) and acyl carrier protein (ACP, Figure 1) play key roles: CoA is involved in the first condensation reaction, and ACP is present in all the pathway intermediates [9]. CoA is constituted of 3′-phosphate adenosine linked to a diphosphate unit, itself bound to a pantetheine unit. ACP is the product of the *acpP* gene and is highly conserved amongst pathogens. In *E. coli*, *Ec*ACP is constituted of (i) seventy-seven amino acids organised in two α-helices and (ii) a pantetheine unit linked to a serine (Ser36 in *E. coli*) through a phosphate group [10]. The bounding serine is always included in an Asp-Ser-Leu motif.

**Scheme 1:**
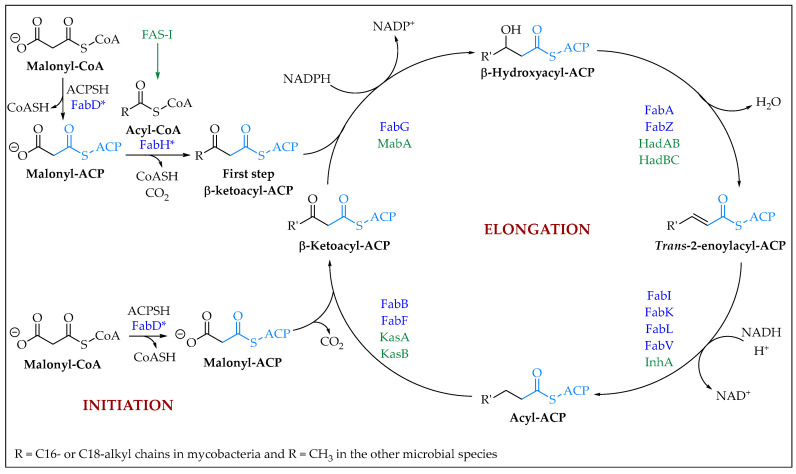
Schematic of type II fatty acid synthase system. Enzymes in green exist in mycobacteria, while those in blue catalyse the FAS-II system in Gram-negative and -positive bacteria and in *Plasmodium* spp. Enzymes labelled with * are involved in the FAS-II system of all pathogens. FAS-II is constituted of an initiation step identical in all pathogens (FabD and FabH) and iterated cycles of reduction (FabG or MabA), dehydration (FabA and FabZ or HadAB and HadBC), reduction (FabI, FabK, FabL and FabV or InhA) and condensation (FabB and FabF or KasA and KasB). In the initiation step of mycobacteria, acyl-CoAs are synthesized by FAS-I.

Among pathogens, FAS-II is identical in Gram-negative and -positive bacteria and *Plasmodium* spp. but slightly different in mycobacteria. Nevertheless, the FAS-II system always consists of an initiation phase and an elongation cycle (Scheme 1). Initially, malonyl-CoA is transferred to ACP by FabD [11]. The elongation cycle is initiated by FabH, which condenses acyl-CoA and malonyl-ACP to form a β-ketoacyl-ACP. In mycobacteria, acyl-CoA consists of C16- to C18-unit chains, while it is an acetyl-CoA in other pathogens [12,13]. In the cycle, the β-ketoacyl-ACP is reduced by FabG (MabA in mycobacteria) in the presence of nicotinamide adenine dinucleotide phosphate (NADPH) [14]. Next, β-hydroxyacyl-ACP is dehydrated by FabA or FabZ (HadAB or HadBC in mycobacteria) to an enoyl acyl-ACP [15,16], which is then reduced by FabI (InhA in mycobacteria), FabK, FabL or FabV depending on several parameters such as chain length or nature of the pathogen [13,17,18,19]. The synthesized acyl-ACP is condensed with malonyl-ACP thanks to FabB or FabF (KasA or KasB in mycobacteria) to produce a β-ketoacyl-ACP elongated with two additional carbons, and the cycle iterates [20,21].

### 2.1. Malonyl-CoA: ACP Transacylase

As previously mentioned, FabD is involved in the initiation step of bacterial fatty acid biosynthesis and catalyses the transfer of a malonyl moiety from malonyl-CoA to ACPs [22,23,24,25].

**Figure 2:**
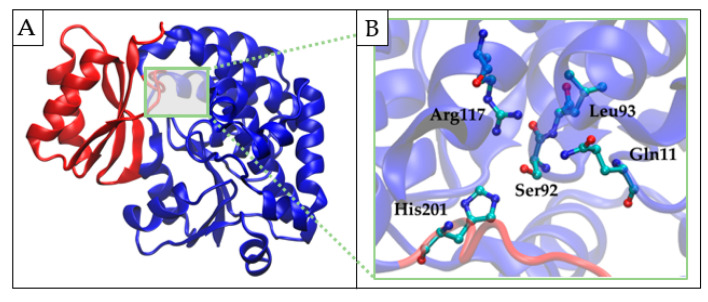
(**A**) *Ec*FabD apo structure (PDB ID 1MLA). The ferredoxin-like and α/β hydrolase subdomains of *Ec*FabD are coloured in red and blue, respectively. Visual molecular dynamics was used to visualise enzymes and their active sites [32], unless otherwise specified. (**B**) View of the apo active site of *Ec*FabD (PDB ID 1MLA). This view is focused on the five catalytic residues.

Crystal structures of FabD from *E. coli* [26], *Acinetobacter baumannii* [27], *Burkholderia pseudomallei* [28] and *Mycobacterium tuberculosis* [29] are available in the PDB. The tertiary fold of FabD is composed of two subdomains: (i) a larger α/β hydrolase subdomain and (ii) a smaller ferredoxin-like subdomain (Figure 2A) [26,30]. The active site of FabD is located within a cleft at the interface between these two subdomains and is constituted of five conserved residues: Arg, Ser, His, Gln and Leu (Figure 2B) [26,30,31]. Several structures of EcFabD are available in the PDB either in apo-form (PDB ID 1MLA) or in complex with different substrates such as malonyl and CoASH (PDB ID 2G2Z), malonate (PDB ID 2G2Y), glycerol (PDB ID 2G1H), sulphate (PDB ID 2G2O) and more recently with AcpP (PDB ID 6U0J) [26,30,31]. Interactions between substrates and the enzyme appearing in these structures sustain the catalytic role of each residue of the active site. In EcFabD, Ser92 is directly implicated in the exchange between -SCoA and -SACP supported by His201, while Arg117, Gln11 and Leu93 ensure correct substrate position.

**Figure 3:**
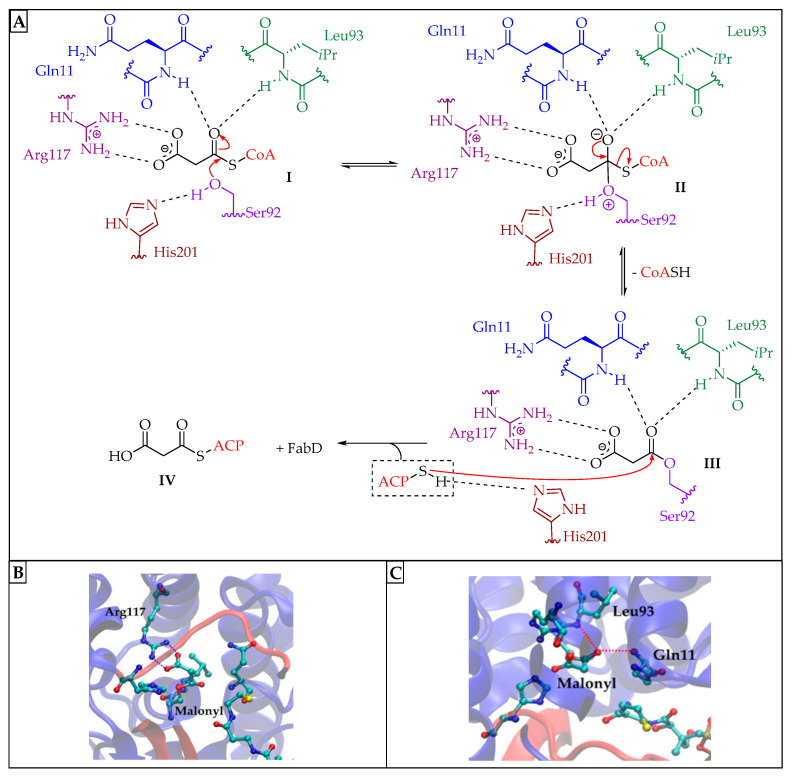
(**A**) Scheme of the mechanism of the transfer of malonyl moiety from malonyl-CoA to ACP catalysed by FabD in *E. coli*. (**B**,**C**) Views of the active site of *Ec*FabD in which malonyl-CoA was crystallized (PDB ID 2G2Z). They highlight (**B**) the bidentate salt bridge (blue dots) between the carboxylate moiety of the malonate and the guanidinium group of the Arg117 and (**C**) hydrogen bonds between the carbonyl of malonate covalently bound to Ser92 and the main-chain amides of Gln11 and Leu93 which form the oxyanion hole (red dots).

The transfer of the malonyl group from malonyl-CoA to ACP occurs in three main steps (Figure 3A). First, His201 activates Ser92 through a hydrogen bond and facilitates the nucleophilic attack on the malonyl-CoA thioester **I** carbonyl. The appearing negative charge on the oxygen atom of malonyl-Ser92 intermediate **II** is stabilised by the oxyanion hole formed by the main-chain amides of Gln11 and Leu93 [30,31]. The structure of *Ec*FadD with processed malonyl-CoA reveals that, in the second step, intermediate **III** covalently binds to Ser92 and forms a bidentate salt bridge between the carboxylate of the malonyl moiety and the guanidinium of Arg117, which is involved in substrate recognition [31]. Orientation of the formed ester bond allows nucleophilic attack by the phosphopantetheine arm of ACP to obtain **IV** [31].

Although FabD is a vital enzyme and a potential target for ATM drug discovery [33,34,35], there is still no established inhibitor of this enzyme.

### 2.2. Condensing Enzymes

#### 2.2.1. Description of FabB, FabF and FabH

Condensing enzymes, such as FabB, FabF and FabH, catalyse the Claisen condensation reaction. They transfer an acyl primer to malonyl-ACP to synthesize β-ketoacyl-ACP elongated with two additional carbons and exist in two varieties [25]. On the one hand, β-ketoacyl-ACP synthase **I** (FabB or KASI) and **II** (FabF or KASII) are components of the elongation cycle. On the other hand, β-ketoacyl-ACP synthase **III** (FabH or KASIII), catalyses the initiation step [36,37,38]. In mycobacteria, the FabB and FabF homologues are termed KasA and KasB, respectively [39,40].

FabB is required in the elongation of unsaturated fatty acids [41]. FabH is the only condensing enzyme playing a key role in the fatty acid biosynthesis. Indeed, as the initiator of elongation, FabH is essential for the biosynthesis [42,43,44]. FabB and FabF both use acyl-ACPs ranging from four to sixteen carbon atoms in length as primers, whereas FabH uses acetyl-CoA in bacteria and *P. falciparum* and FAS-I acyl-CoA in mycobacteria [36,45,46]. 

**Figure 4:**
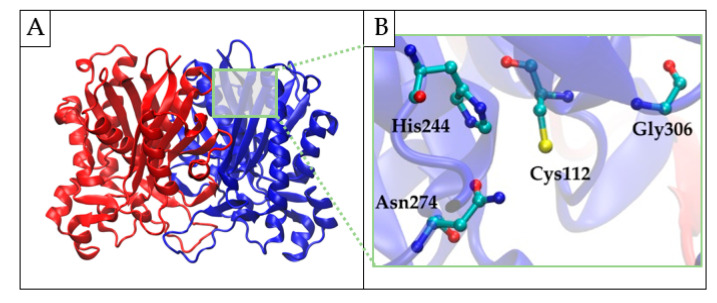
(**A**) *Ec*FabH apo dimer structure (PDB ID 1HN9). One FabH monomer is coloured in blue, the other in red. (**B**) View of the active site of *Ec*FabH (PDB ID 1HN9). This view is focused on the four catalytic residues.

FabB and FabF are approximately 40% identical at the primary sequence level and have less than 10% sequence homology with FabH [23,47,48]. Nevertheless, the three *E. coli* KAS isozymes are all dimers in which each monomer possesses an α/β/α/β/α fold (Figure 4A) [49]. Architectures of their active sites are similar and consist of a cysteine and two residues with hydrogen-bonding potency (Figure 4B). The active sites of FabB/F include a Cys-His-His triad: Cys163-His298-His333 (*Ec*FabB) and Cys163-His303-His340 (*Ec*FabF). *Ec*FabH has a slightly different catalytic triad which comprises Cys112, His244 and Asn274 [36,42,50,51]. The catalytic triad of the FAS-I ketoacyl synthase domain, responsible for the condensation reaction, is identical to those of FabB (with a Cys1305-His1542-His1583 triad in the yeast *Saccharomyces cerevisiae*) [25,52].

The three-dimensional structures of *Ec*FabB (PDB IDs 1DD8, 1H4F, 1EK4, 5KOF and 6OKC) [49,53,54,55,56], *Ec*FabF (PDB IDs 1KAS, 7L4E and 6OKC) [56,57,58] and *Ec*FabH (PDB IDs 3IL9 and 2GYO) [59,60] were solved with or without substrates. In *Ec*FabH, Cys112 behaves like a nucleophile towards the thioester group, while the backbone amides of Gly306 and Cys112 stabilize the oxyanion appearing during the transition state of acetyl transfer. Arg36 and Asn247 also play an important role and ensure correct substrate position (not shown in Figure 4) [61].

**Figure 5:**
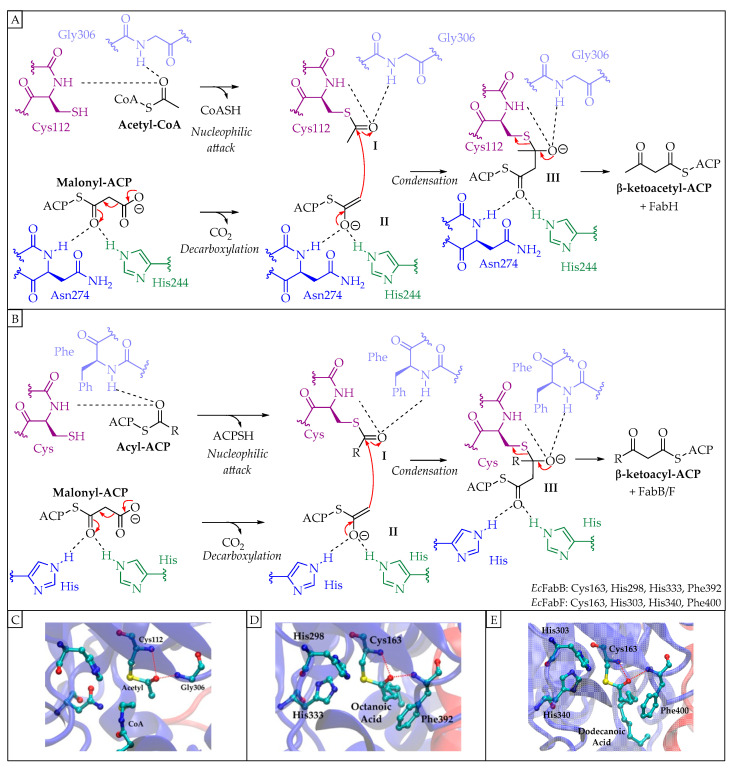
Scheme of the mechanism of the condensation reactions catalysed by FabH (**A**) and FabB/F (**B**) in *E. coli*. (**C**) View of the active site of *Ec*FabH-acetyl-CoA structure (PDB ID 1HNH) highlights hydrogen bonds between the carbonyl of acetyl covalently bound to Cys112 and the main-chain amides of Cys112 and Gly306 which form the oxyanion hole (red dots). (**D**) View of the active site of *Ec*FabB-octanoic acid structure (PDB ID 2BUI) highlights hydrogen bonds between the carbonyl of octanoic acid covalently bound to Cys112 and the main-chain amides of Cys163 and Phe392 which form the oxyanion hole (red dots). (**E**) View of the active site of *Ec*FabF-dodecanoic acid structure (PDB ID 2GFY) highlights hydrogen bonds between the carbonyl of dodecanoic acid covalently bond to Cys163 and the main-chain amides of Cys163 and Phe400 which form the oxyanion hole (red dots).

The Claisen condensation, catalysed by the condensing enzymes, is tripartite and very similar for all of the enzymes. It will be described more precisely for *Ec*FabH, in which the four main amino acids involved are Cys112, His244, Asn274 and Gly306 (Figure 5A,C). In the first step, the thioester group of the incoming acetyl-CoA undergoes a nucleophilic attack by the sulfhydryl group of Cys112 to form a thioacetyl enzyme intermediate **I**, while CoA is released. In the meantime, malonyl-ACP is decarboxylated and leads to enolate intermediate **II**, which is stabilised via hydrogen bonds by Asn274 and His244 [46]. In the second step, a nucleophilic attack on the thioester group of **I** by the carbanion of the enolate intermediate **II** occurs. An oxyanion hole, formed by the amide groups of Cys112 and Gly306 [46,61], stabilises the transient tetrahedral intermediate **III**, which finally yields β-ketoacyl-ACP [46,62]. In FabB and FabF (Figure 5B,D,E), the hydrogen bond donors are two histidines instead of one histidine and one asparagine in FabH. Moreover, the decarboxylation is promoted by a phenylalanine (Phe392 or Phe400) instead of a glycine (Gly306).

Among the condensing enzymes, FabH is the most studied because (i) it is ubiquitous in pathogens [37], and (ii) its 3D structure and (iii) its functions are highly conserved in many human pathogens [43,63,64]. Contrary to FabB, the amino acid sequence of the FabH active site differs from those of the catalytic domain of the mammalian FAS-I responsible for the same elongation step. Thanks to better selectivity, ATM drugs targeting FabH should limit side effects in humans [25,43,52,63,65]. 

#### 2.2.2. FabB, FabF and FabH Inhibitors

##### Benzoic Acids

**Figure 6:**
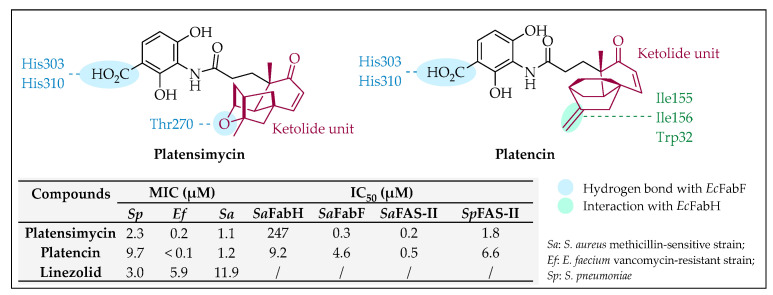
Structures, in vitro activities and representation of main interactions with *Ec*FabF/H of **platensimycin** and **platencin** [66,69].

**Platensimycin** and **platencin** (Figure 6) are two natural products isolated from *Streptomyces platensis* MA7339 [21,66]. While both possess a 3-amino-2,4-dihydroxy benzoic acid core, their ketolide units differ with a tetrahydropyran ring for **platensimycin** and a methylenecyclohexane core for **platencin**. Both **platensimycin** and **platencin** show potent broad-spectrum activity against Gram-positive bacteria with minimal inhibitory concentrations (MICs) close to those of linezolid against *S. aureus* (methicillin-sensitive strain), *Enterococcus faecium* (vancomycin-resistant strain) and *S. pneumoniae* [66]. **Platencin** and **platensimycin** do not exhibit cross-resistance with methicillin, vancomycin, linezolid or macrolides [21,66,67]. Besides, **platensimycin** and **platencin** were efficiently used in a murine model of common *S. aureus* infection and no toxicity was observed [21,66]. Whole-cell experiments demonstrated that these two natural products inhibit the fatty acid biosynthesis of *S. aureus* (MIC ≈ 1 µM) and *S. pneumoniae* (MIC = 2–10 µM). **Platensimycin** preferentially targets FabF (half inhibitory concentration (IC_50_) against *Sa*FabF of 0.3 µM) compared to FabH (IC_50_(*Sa*FabH) = 247 µM) while **platencin** inhibits FabF and FabH activities in the micromolar range [66]. This difference in activity could be explained by the interactions formed between **platensimycin** and **platencin** and the enzymes. Indeed, docking studies carried out by Singh et al. with *Ec*FabF [68] and Jayasuriya et al. with *Ec*FabH [69] showed that carboxylic acid groups of **platensimycin** and **platencin** create the same hydrogen bonds with His303 (part of the active site) and His310 of *Ec*FabF. However, **platensimycin** favours interaction with *Ec*FabF through hydrogen bonding between Thr270 and its tetrahydropyran ring, while **platencin** interacts preferably with *Ec*FabH, creating a link between the triad Ile155-Ile156-Trp32 and the methylenecyclopentane.

**Figure 7:**
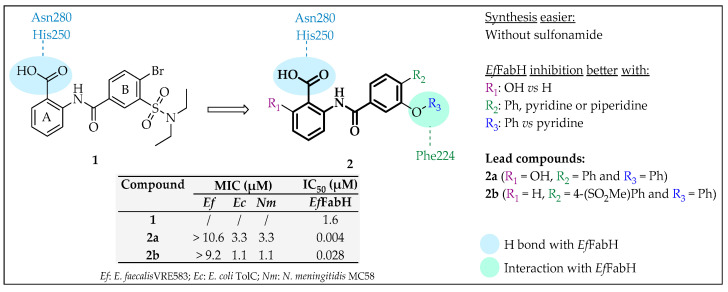
Structures, SAR and representation of main interactions with *Ef*FabH of benzoylaminobenzoic acids **1** and **2** and in vitro activities of lead compounds [70].

In 2005, a team of Quorex Pharmaceuticals, through a structure-based drug design approach on commercial compounds, selected two thousand five hundred potent FabH inhibitors [70]. Among them, benzoic acid **1** (Figure 7) was chosen for optimisation, and SAR was carried out through the structural analysis of the **1**-*Ef*FabH complex. Two main hydrogen bonds were highlighted between carboxylate oxygens of **1** and two amino acids of the active site (His250 and Asn280). Forty-five analogues **2** (Figure 7) were synthesized, modulating ring A and B substituents. To facilitate synthesis of *para*-substituted compounds and to create an interaction with Phe224, the sulphonamide was replaced by a phenoxy or oxypyridine group. The SAR study suggested that (i) a hydroxy group at the *ortho*-position of carboxylic acid in ring A (R_1_), (ii) a phenyl ring or weakly basic substituents such as pyridine or piperidine at the *para*-position of ring B (R_2_) and (iii) a phenoxy instead of a sulphonamide (R_3_) increase the inhibitory activity of **2**. For lead compounds **2a** and **2b**, no ATM activity was observed against *Enterococcus faecalis*, but they displayed good activities against *E. coli* and *Neisseria meningitidis*.

##### Five-Membered Heterocycles

**Figure 8:**
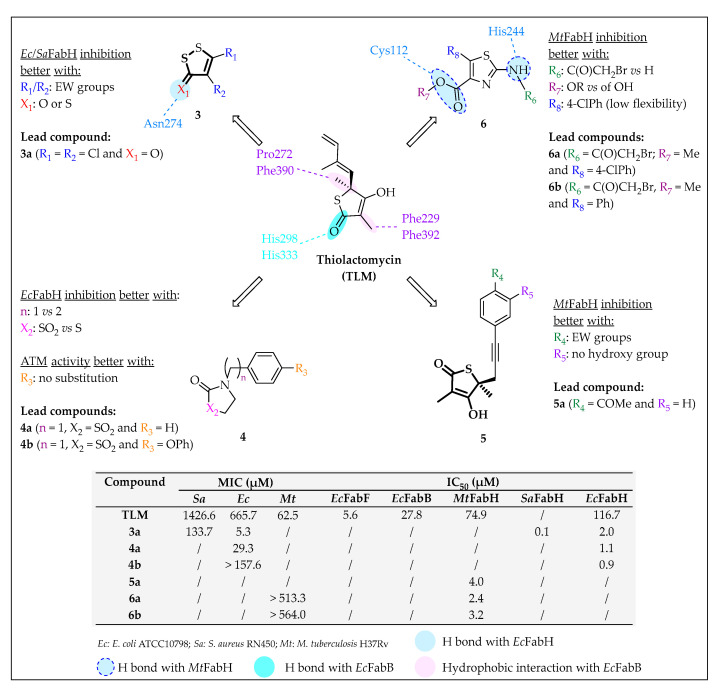
Structures, SAR and representation of main interactions with *Ec*FabB, *Ec*FabH and *Mt*FabH of **TLM** and its analogues **3–6** and in vitro activities of lead compounds [41,65,76,77].

**Thiolactomycin** (**TLM**, Figure 8) is another natural product isolated in 1981 from the strain No. 2-200 of *Nocardia* extracted from a soil sample of Sayama City in Japan [71]. **TLM** is more active against Gram-positive bacteria [71] and some Gram-negative anaerobes [72]. Furthermore, **TLM** inhibits mycolic acid biosynthesis and, consequently, mycobacteria [73]. This molecule reversibly inhibits the FAS-II system but not FAS-I [74]. While it displays weak *Ec*FabH inhibition, **TLM** is more active against *Ec*FabB, *Ec*FabF, *Sp*FabH and *Hi*FabH [36,41]. In 2001, Price et al. showed several key interactions through structural analysis of the *Ec*FabB–**TLM** binary complex: (i) two methyl groups of **TLM** are nestled within two hydrophobic pockets comprising either the couple Phe229/Phe392 or Pro272/Phe390, and (ii) the carbonyl oxygen of **TLM** is involved in hydrogen bonds with the two histidines of the active site (His298 and His333) [41,75].

In 2004, the team of Reynolds screened almost one hundred and twenty thousand compounds possessing **TLM** structural characteristics from the National Cancer Institute database to develop more potent FabH inhibitors. They identified two sets of interesting compounds: (i) substituted 1,2-dithiol(e)-3-(thi)ones (**3**, Figure 8) [76] and (ii) thiazolidin-2-ones (**4**, Figure 8) [65]. In the first study, SAR analysis with fifteen compounds **3** was carried out, modulating both the 4,5-positions (R_1_ and R_2_) of 1,2-dithiol(e)-3-(thi)one with aromatic ring, halogen atoms or alkyl chains and studying the influence of either carbonyl or thiocarbonyl group (X_1_) in 3-position on the FabH inhibition. It resulted in five hits (IC_50_(*Ec*FabH) < 10 µM), including the lead compound **3a**. The SAR analysis suggested that (i) electro-withdrawing (EW) groups in R_1_ and/or R_2_, especially chlorine atom, allow efficient inhibition of *Sa*FabH and *Ec*FabH and that (ii) carbonyl and thiocarbonyl groups possess the same potency. The lead compound **3a** displayed better ATM activities against *E. coli* and *S. aureus* and was sixty times more effective than **TLM** against *Ec*FabH (IC_50_ = 2.0 vs. 116.7 µM). A docking study of **3a** with *Ec*FabH showed that a strong hydrogen bond was created between the carbonyl group and Asn274. In the second study, Alhamadsheh and co-workers modulated **4** [65] and synthesized twenty-one thiazolidin-2-ones. The SAR study focused on the *N*-substitution and the variation of the oxidation state of the sulphur group (X_2_). Four hits were identified (IC_50_(*Ec*FabH) < 10 µM), including **4a** and **4b**, and some SAR were highlighted: (i) the decrease in the oxidation state of the sulphur moiety leads to high loss of *Ec*FabH inhibition, (ii) the nitrogen atom must be benzyled (n = 1), and (iii) the substitution at the *para*-position of the benzyl (R_3_) cancels the ATM activity but not the inhibitory activity.

In 2004, to develop *Mt*FabH inhibitors, Senior et al. designed seven **TLM** analogues **5** (Figure 8) by modulating alkyne phenyl substituents (R_4_ and R_5_) with nitro, cyano, hydroxy or ketone groups [77]. They observed that the *meta*-hydroxy group (R_5_) decreased the inhibitory activity against *Mt*FabH. Nevertheless, compounds with *para*-EW substituents (R_4_) displayed highly improved activities. The best activity (**5a**) was obtained with the *para*-acetyl group (IC_50_ = 4.0 vs. 74.9 µM for **TLM**). Unfortunately, the ATM activities of **5** were not evaluated.

In 2009, Al-Balas et al. tried to develop simplified analogues of **TLM** and designed and synthesized sixteen 2-aminothiazole-4-carboxylate derivatives **6** (Figure 8) as potent *Mt*FabH inhibitors [78]. The aminothiazole core was substituted on (i) the 2-position with amines or bromoacetamides (R_6_), (ii) the 4-position (R_7_) with esters or carboxylic acids, and (iii) the 5-position with alkyl chains or aromatic rings (R_8_). Unfortunately, many of these compounds (including the lead compounds **6a** and **6b**) did not display ATM activity against *M. tuberculosis*. The SAR study showed that (i) an ester instead of carboxylic acid at position 4 of 2-aminothiazole (R_7_) and (ii) a phenyl group at position 5 of 2-aminothiazole with or without a *para*-chlorine atom (R_8_) encourage the inhibitory activity. The lead compound **6a** inhibited the enzyme with IC_50_ of 2.4 µM and displayed no cytotoxicity against HS-27 human fibroblast cells at 100 µM. Moreover, it did not inhibit FAS-I. Docking studies highlighted two hydrogen bonds with amino acids of the active site between (i) the secondary amine and His244 and (ii) the carbonyl at position 4 and Cys112.

**Figure 9:**
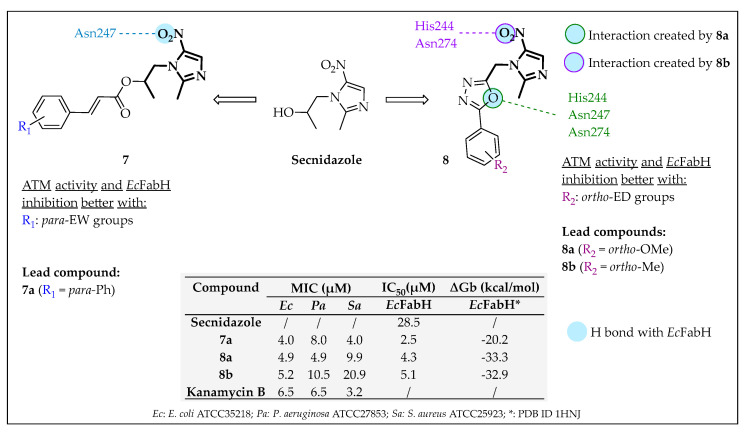
Structures, SAR and representation of main interactions with *Ec*FabH of **secnidazole** derivatives **7** and **8** and in vitro activities and ΔGb of lead compounds [79,80].

Several teams were inspired by **secnidazole** (Figure 9), a nitroimidazole antibacterial (ATB) drug, to develop FabH inhibitors as **secnidazole** analogues based on a cinnamic acid scaffold (**7**, Figure 9) [79] or on an oxadiazole core (**8**, Figure 9) [80]. Zhang et al. synthesized twenty cinnamic acid ester derivatives **7**, and Li et al. synthesized eighteen oxadiazoles **8**. Both teams modulated phenyl substituents. They obtained seven hits, including lead compounds **7a**, **8a** and **8b**, which were five- to six-fold more potent than **secnidazole** against *Ec*FabH. The SAR study on **7** revealed that EW groups at the *meta*- or *para*-position lead to improvement in both ATM activity and *Ec*FabH inhibition. On the contrary, in compounds **8**, electro-donating (ED) substituents in *ortho* were favourable to display ATB activities against Gram-positive and -negative bacteria and to inhibit *Ec*FabH. Docking calculations between lead compounds and *Ec*FabH were consistent with inhibitory activity (better binding enthalpy ΔGb for lead compounds). The binding model with *Ec*FabH showed that the nitro oxygen of **7a** creates a hydrogen bond with Asn247, whereas those of **8b** interact with His244 and Asn274. In **8a**, the nitro group is not involved in any interaction, but the oxadiazole oxygen forms hydrogen bonds with two amino acids of the active site (His244 and Asn274) and Asn247.

**Figure 10:**
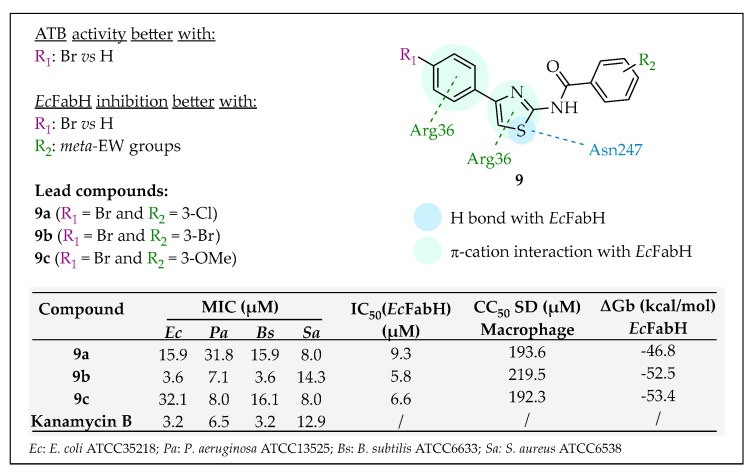
Structure, SAR and representation of main interactions with *Ec*FabH of thiazole derivatives **9** and in vitro activities and ΔGb of lead compounds [81].

In 2014, Li et al. described thiazole derivatives containing benzamide group **9** (Figure 10) as potent *Ec*FabH inhibitors [81]. They synthesized twenty-four compounds and focused on 4-phenyl (R_1_) and 2-benzamide (R_2_) substitutions. Most of them exhibited ATM activities against *E. coli*, *P. aeruginosa*, *Bacillus subtilis* and *S. aureus*, and three compounds (**9a–c**, Figure 10) possessed IC_50_ values lower than 10 µM against *Ec*FabH. Particularly, the broad-spectrum activity of **9b** (MIC = 3.6–14.3 µM against the four strains) was comparable to that of **kanamycin B**. SAR study showed that (i) *para*-bromine (R_1_) substitution of the 4-phenyl results in better ATB and inhibitory activities and (ii) compounds with EW groups in *meta* of the benzamide (R_2_) display better *Ec*FabH inhibition. Moreover, these compounds displayed weak cytotoxicity against human macrophage. A docking study was relevant with inhibitory activity, since **9a–c** possessed the lower ΔGb of the series. The binding model of **9b** and *Ec*FabH showed that three main interactions are formed: two π-cations between both the thiazole or the phenyl rings and Arg36 and one hydrogen bond between the sulphur atom on the thiazole ring and Asn247. In the *Ec*FabH functioning, these two amino acids ensure correct position of the natural substrate.

##### Fused Cycles

**Figure 11:**
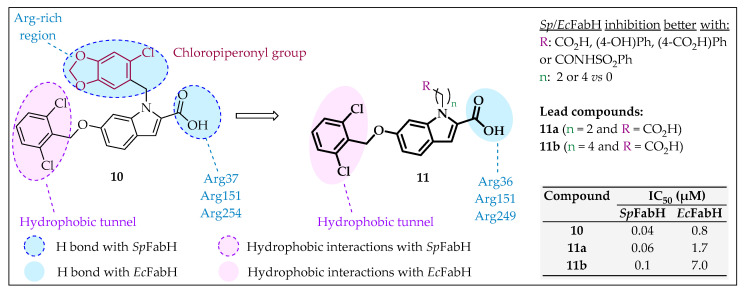
Structures, SAR and representation of main interactions with *Sp*FabH and *Ec*FabH of indole analogues **10** and **11** and in vitro activities of lead compounds [42].

In 2003, Daines et al. identified indole-2-carboxylic acid **10** (Figure 11) as a potent *Sp*FabH inhibitor via high-throughput screening [42]. Unfortunately, co-crystallization of **10** with either *Sp*FabH or *Ec*FabH was impossible due to its poor hydrosolubility. Hence, a homology model of *Sp*FabH using *Ec*FabH-CoA co-crystal as structural template was built for the docking of **10**. In this predicted binding mode, the main interactions were highlighted: (i) the carboxylic acid of **10** binds to the arginines on the protein surface (Arg37, Arg151 and Arg254, located at the top of the active site), (ii) the 2,6-dichlorobenzyl group interacts in the hydrophobic tunnel of the active site, and (iii) the 6-chloropiperonyl group is located near the arginine-rich region on the top of the enzymatic tunnel. Thus, analogues with more polar side chains instead of lipophilic chloropiperonyl groups (R and n) were designed to improve both the inhibitory activity and the aqueous solubility of this indole family. Seven indole-2-carboxylic acid analogues **11** including a polar group at the 1-position of the indole (R), preferably carboxylic acid function carried by aryl group or alkyl chain, were synthesized and evaluated. Unfortunately, these compounds lacked ATM activity, and none of them displayed better inhibitory activity than **10**. Nonetheless, the increase in inhibitor hydrosolubility allowed the crystallisation of the first complex between synthetic small structure **11b** and *Ec*FabH. The resulting information was consistent with the interactions previously highlighted using the homology model. 

**Figure 12:**
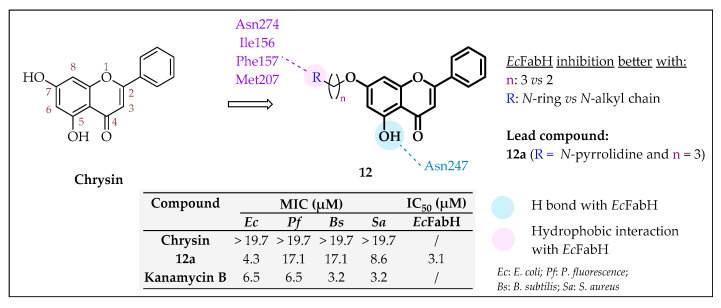
Structures, SAR and representation of main interactions with *Ec*FabH of **chrysin** and its analogues **12** and in vitro activities of lead compounds [43].

In 2009, **chrysin** analogues **12** (Figure 12) were designed by Li and co-workers as *Ec*FabH inhibitors [43]. They synthesized eighteen compounds with different R groups and spacer length (n) grafted at C7-position of the chromen-4-one core. All of them exhibited ATM activities against both Gram-positive (*B. subtilis* and *S. aureus*) and -negative (*E. coli* and *Pseudomonas fluorescence*) bacteria, but only three displayed IC_50_(*Ec*FabH) lower than 10 µM, including the lead compound **12a**. SAR analysis showed that (i) three- is more favourable than two-carbon spacer (n), (ii) the non-aromatic *N*-heterocyclic ring at 7-position (R) exhibits higher potencies than **chrysin**, and (iii) alkyl amines instead of *N*-heterocyclic rings decrease inhibitory activity. The lead compound **12a** had broad-spectrum activity, close to the reference **kanamycin B**, correlating with its good inhibitory activity against *Ec*FabH (IC_50_ = 3.1 µM). Molecular docking between **12a** and *Ec*FabH revealed that the 5-hydroxy group forms a hydrogen bond with Asn247 in the active site, while the pyrrolidine moiety at C7-position can establish a hydrophobic interaction with Asn274, Ile156, Phe157 and Met207. 

**Figure 13:**
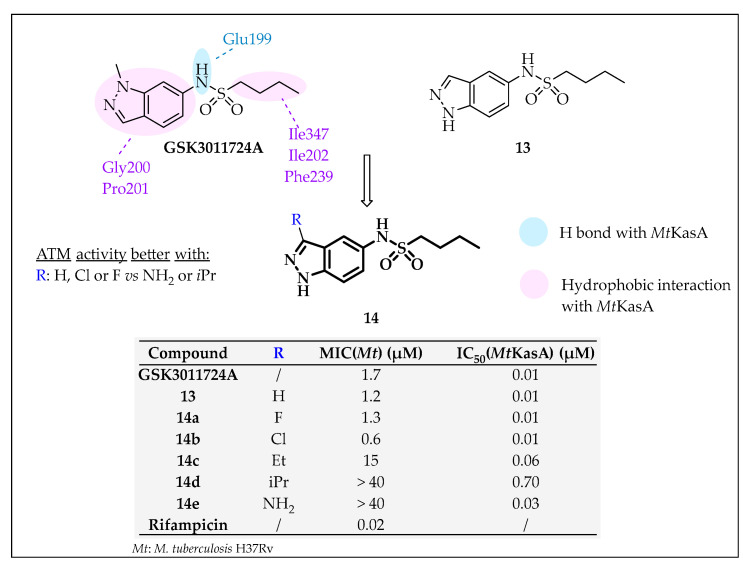
Structures, SAR and representation of main interactions with *Mt*KasA of **GSK3011724A** and its derivatives **13–14** and in vitro activities of lead compounds [83,84,85].

**GSK3011724A** (Figure 13) or *N*-(1-methyl-1*H*-indazol-6-yl)butane-1-sulphonamide was discovered via a phenotypic screening campaign against *M. tuberculosis* realised by GlaxoSmithKline in 2013 amongst two hundred twenty-eight molecules [82]. In 2016, **GSK3011724A** was identified as an *Mt*KasA inhibitor by Abrahams et al. [83]. The analysis of the *Mt*KasA-**GSK3011724A** co-crystal structure revealed that (i) the aliphatic tail of **GSK3011724A** mimics binding of the natural substrate in a hydrophobic pocket composed of Ile347, Ile202 and Phe239 as main amino acids, (ii) the indazole ring lies in a channel formed by Gly200 and Pro201, and (iii) the amine of the sulphonamide forms a hydrogen bond with Glu199 [83,84,85]. This compound exhibited ATM activity against *M. tuberculosis* (MIC = 1.7 µM) correlated with inhibitory activity against *Mt*KasA (IC_50_ = 0.01 µM) without cytotoxicity on HepG2 cell lines (IC_50_ > 100 µM) [84]. Moreover, **GSK3011724A** was active in vivo in an acute mouse model (ED99 = 38 mg/mL). Later, in 2020, Cunningham et al. identified a new indazole sulphonamide **13**, which is structurally similar to **GSK3011724A**, with slightly improved in vivo potency related to its better microsomal stability (Cli(mouse) = 2.4 vs. 6.1 mL/min/g) [84]. The sulphonamide function of both **GSK3011724A** and **13** is necessary for activity, as shown in the docking study, but unfortunately, it is also the parent of a toxic aniline metabolite formed, making them unsuitable for future clinical trials. To avoid this mutagenic behaviour, SAR investigation was carried out with **13** as lead compound [84]. More than ninety-seven compounds with the sulphonamide function were synthesized but all possessed mutagenic properties. However, one series of compounds **14** (Figure 13) was brought to light due to its important inhibitory activity, which often correlated with ATB potency (**14a–c**). Additionally, SAR study demonstrated that (i) the sulphonamide is required, and (ii) butyl sulphonamide is favoured for inhibitory activity, as for **GSK3011724A** and **13a**, but (iii) small substituents such as halogen atoms at the 3-position of the indazole (R) are well tolerated and give more active compounds against *M. tuberculosis* (**14a–b**). Among the synthesized analogues, only nine were active against *M. tuberculosis*. However, MIC and IC_50_ values were not always correlated, as some compounds without ATM activity exhibited *Mt*KasA inhibitory activity with an IC_50_ below 1 µM (**14d** and **14e**).

##### Others

**Figure 14:**
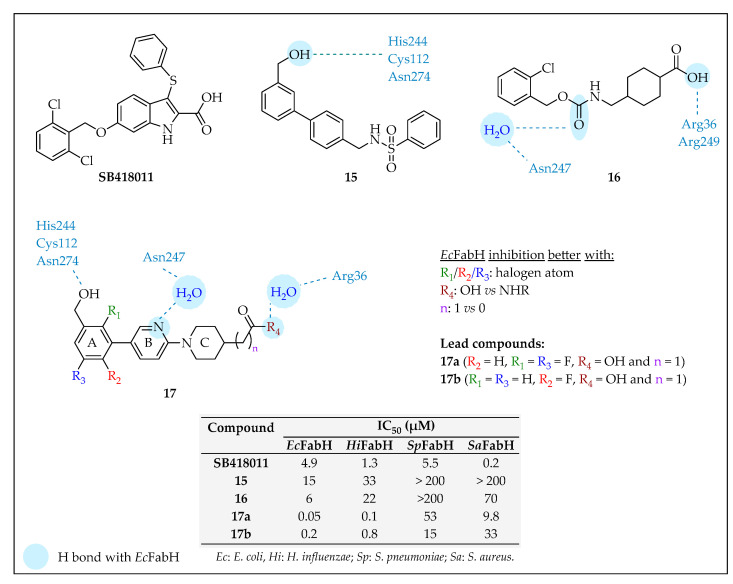
Structures and representation of main interactions with *Ec*FabH of **SB414011**, **15–17** and in vitro activities of lead compounds [36,86].

Two AstraZeneca compound collections were screened to identify potent *Ec*FabH inhibitors. Thanks to that, three hits were obtained (**SB418011** and **15–16**, Figure 14), among which **SB418011** was the most active (IC_50_ = 0.2–5.5 µM against *Ec*FabH, *Hi*FabH, *Sp*FabH and *Sa*FabH) [36,86]. Unfortunately, no ATB activity was evaluated for these compounds, which did not allow a correlation with enzymatic assays. In parallel, co-crystal structures of *Ec*FabH with **15** and **16** were obtained. Structural analysis of these models highlighted several hydrogen bonds between the hydroxy function of **15** and three amino acids of the catalytic site of *Ec*FabH (His244, Cys112 and Asn247), or for **16** between (i) the amide carbonyl function and Asn247 via a water molecule and (ii) the hydroxy group of the carboxylic function and Arg36 and Arg249. Based on these observations, twenty new hybrids **17** (Figure 14) were designed by merging the biaryl moiety of **15** and the acidic site of **16** to study in particular the influence of phenylethanol substitutions (R_1–3_) [86]. Thus, the SAR studies underlined that, in order to inhibit *Ec*FabH, the following modifications are the most favourable: (i) halogen atoms in R_1_, R_2_ or R_3_ positions of ring A, (ii) more flexibility (n = 1 vs. 0) and (iii) a carboxylic acid function instead of amide (R_4_ = OH vs. NHR). The lead compounds **17a** and **17b** displayed better activities against *Ec*FabH than the three hits **15**, **16** and **SB418011**. They were three hundred (**17a**) and seventy-five (**17b**) folds more active than **15** and one hundred and twenty (**17a**) and thirty (**17b**) folds more active than **16**. As expected, structural analysis highlighted that the coupling of the two pharmacophores of **15** and **16** within a single compound resulted in a greater affinity for *Ec*FabH due to a cumulative effect of the interactions independently observed for **15** and **16**. Thus, hydrogen bonds are established between (i) the hydroxy group of the phenylethanol moiety and the three amino acids of the active site (Cys112, His244 and Asn274), (ii) the carboxylic acid and Arg36 via a water molecule, and (iii) the pyridine nitrogen and Asn247 via a water molecule.

**Figure 15:**
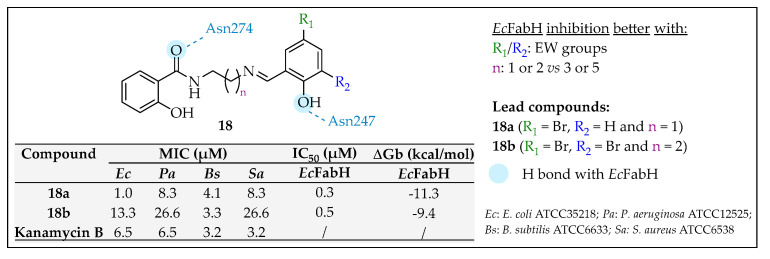
Structure, SAR and representation of main interactions with *Ec*FabH of Schiff bases **18** and in vitro activities of lead compounds [87].

Schiff bases are compounds of interest to develop FabH inhibitors. In 2009, Cheng et al. developed Schiff bases **18** with potent ATM activities based on molecular modelling and SAR studies (Figure 15) [87]. Forty-eight compounds **18** were synthesized with variable carbon-chain lengths between the aromatic rings and different groups (R_1_ and/or R_2_) on the aromatic core of the benzylimine moiety. *Ec*FabH inhibition was increased by the presence of halogen atoms at R_1_ and/or R_2_ instead of hydrogens and a carbon chain of one or two carbon atoms (n = 1 or 2). Furthermore, the lead compounds **18a** (IC_50_(*Ec*FabH) = 0.3 µM) and **18b** (IC_50_(*Ec*FabH) = 0.5 µM) displayed broad-spectrum ATB activities against Gram-negative and -positive bacteria. The binding model realised with **18a** and *Ec*FabH showed that hydrogen bonds are established between the carbonyl oxygen atom of **18a** and Asn274 (part of the active site) and the hydroxy group of the benzylimine and Asn247.

**Figure 16:**
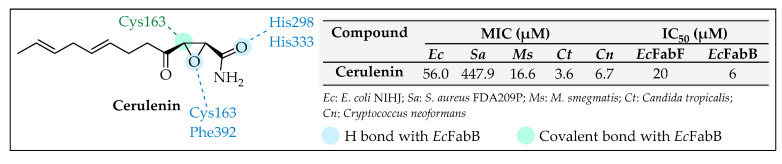
Structure, representation of main interactions with *Ec*FabB and in vitro activities of **cerulenin** [41].

**Cerulenin** (Figure 16) was the first FabB/F inhibitor identified. It was isolated in 1960 from the fungus *Cephalosporium caerulens* [88]. **Cerulenin** displayed growth inhibition against Gram-negative bacteria (MIC = 56.0 µM against *E. coli*) and mycobacteria (MIC = 16.6 µM against *M. tuberculosis*) but weaker activities against Gram-positive bacteria (MIC = 447.9 µM against *S. aureus*). This molecule also possesses antifungal activity (MIC = 3.6 and 6.7 µM against *Candida tropicalis* and *Cryptococcus neoformans* respectively) [88]. **Cerulenin** inhibits both FabB and FabF of *E. coli* (IC_50_ of 6 and 20 µM, respectively) [41] and also the condensation reaction catalysed by the FAS-I system, which could lead to side effects [89]. A docking study with *Ec*FabB showed that hydrogen bonds are created between the active site amino acids (His298 and His333) and the amide oxygen of **cerulenin** and between its epoxide oxygen and Cys163 and Phe392 [41]. In *E. coli*, it was shown that **cerulenin** irreversibly binds with Cys163 of the active site through an S-C link that is induced following the epoxide ring opening of **cerulenin**, which impairs the natural FabB/F/H substrate fixation to form the intermediate **I** (Figure 5) [20,90,91].

#### 2.3. β-Ketoacyl-ACP Reductase

##### 2.3.1. Description of FabG

As mentioned in the introduction, FabG, the β-ketoacyl-ACP reductase, is involved in the first step of the elongation cycle of fatty acid biosynthesis and catalyses the NADPH-dependent reduction of β-ketoacyl-ACP [23,50,92,93,94]. The FabG homologue is labelled MabA. FabG is active on β-ketoacyl-ACPs with acyl chain lengths ranging from four to ten carbons [95]. This reductase is essential for all pathogens and is highly conserved and widely expressed across the pathogenic genome [33,93,96,97]. It is part of the short-chain dehydrogenase/reductase (SDR) family [45].

**Figure 17:**
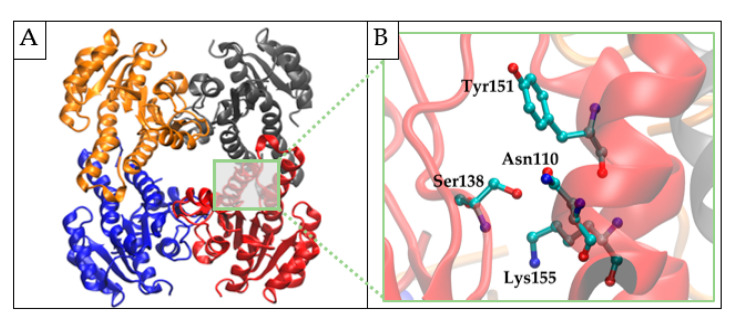
(**A**) *Ec*FabG apo tetramer structure (PDB ID 1I01). The FabG monomers are coloured in blue, red, grey and orange. (**B**)View of the active site of *Ec*FabG (PDB ID 1I01). This view is focused on the four catalytic residues.

Several crystal structures of FabG are available in the PDB from *E. coli* (PDB ID 1I01) [41], *A. baumannii* (PDB ID 6T65) [98], and *P. aeruginosa* (PDB ID 4AG3) [94], for example. In solution, its active quaternary structure is homotetramer (Figure 17A). The tertiary structure of each monomer shows a central twisted β-sheet composed of seven β-strands and surrounded by a total of eight α-helices on both sides. This structure is characteristic of Rossmann fold, which presents a cleft forming a nucleotide binding domain to receive the cofactor [45,47,99]. FabG possesses the Tyr-Lys-Ser catalytic triad (Figure 17B) common to several enzymes belonging to the SDR family. Price et al. determined the crystal structure of *Ec*FabG without cofactor [99] and in complex with the oxidised cofactor NADP^+^ as well as the structure of FabG [Y151F] mutant in complex with the reduced cofactor NADP(H) [100]. These three structures demonstrate the conformational rearrangements occurring upon cofactor binding. In *Ec*FabG, Ser138 ensures correct substrate position, while Tyr151 is directly implicated in the reduction step, and both Lys155 and Asn110 are involved in proton relay.

**Figure 18:**
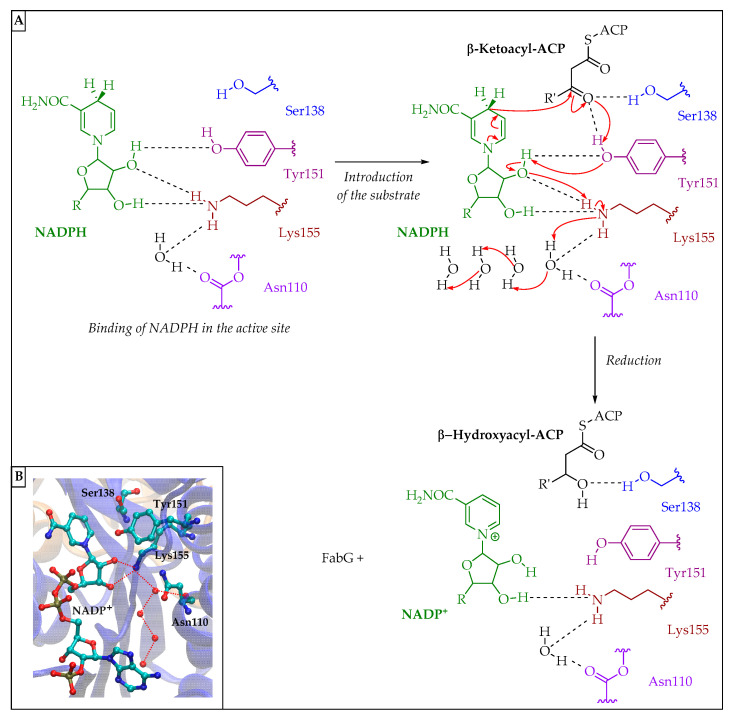
(**A**) Scheme of the mechanism of the reduction catalysed by *Ec*FabG. (**B**) View of the active site of *Ec*FabG-NADP^+^ structure (PDB ID 1Q7B) highlights the hydrogen bonds between the hydroxy groups of NADP^+^ and Lys155 as well as the proton relay system (red dots) involving Lys155, Asn110 and four water molecules shown as red spheres.

The catalytic mechanism can be described as follows (Figure 18). In a preliminary step, the NADPH cofactor binds into the active site via hydrogen bonds with Tyr151 and Lys155 and induces conformational changes, allowing the substrate binding. To be reduced, β-ketoacyl-ACP forms two hydrogen bonds with Ser138 and Tyr151. Then, NADPH donates a hydride to the C3 carbon substrate of β-ketoacyl-ACP, from Tyr151, and a proton is transferred to the oxygen linked to C3 to form the β-hydroxyacyl-ACP. Tyr151 then recovers its proton through a proton relay system which involves Lys155, Asn110, and four water molecules.

The Ser-Tyr-Lys triad residues of FabG are identified in the ketoreductase active site of yeast *Saccharomyces cerevisiae* FAS-I as Ser827-Tyr839-Lys843 [52].

The absence of known isozyme suggests that FabG could be a potential target for developing broad-spectrum ATBs.

##### 2.3.2. FabG Inhibitors

**Figure 19:**
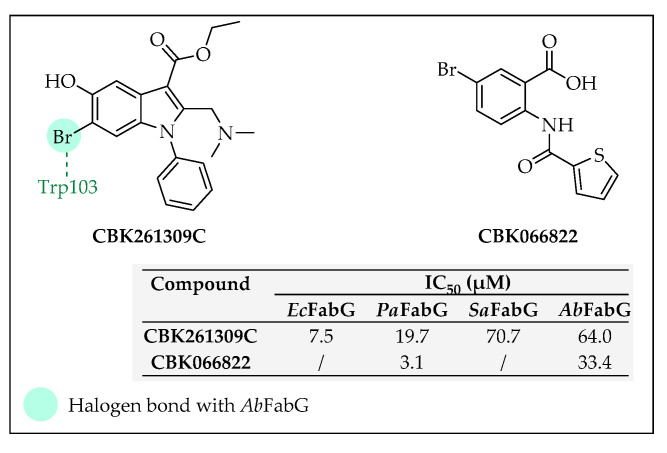
Structures, representation of main interaction with *Ab*FabG and in vitro activities of **CBK261309C** and **CBK066822** [98].

In 2021, Vella et al. highlighted two hits, **CBK261309C** and **CBK066822** (Figure 19), via small-molecule screening as potential FabG inhibitors [98]. The activity of FabG in the presence of thirty-three thousand compounds was assessed by following the formation of NADP^+^. Among these compounds, only one hundred and thirty-one reached the experimental assays with in silico pan assay interference compound filters. These led to thirty-six compounds which were evaluated at a single concentration against eight orthologues of the FabG panel. The hits **CBK261309C** and **CBK066822** were screened against the FabG enzyme of six pathogens: *A. baumannii*, *Salmonella typhimurium*, *E. coli*, *K. pneumoniae*, *P. aeruginosa* and *S. aureus*. While **CBK261309C** acted as a broad-spectrum FabG inhibitor with IC_50_ values in the range of 7.5–70.7 µM depending on pathogens, **CBK066822** exhibited IC_50_ values lower than 100 µM for only two enzymes (*Pa*FabG and *Ab*FabG). **CBK261309C** was more active against *Ec*FabG, whereas **CBK066822** displayed better activity against *Pa*FabG. Unfortunately, no ATM activity was evaluated. Analysis of the co-crystal structure of *Ab*FabG-**CBK261309C** revealed that the bromine atom of the inhibitor and Trp103 form a halogen bond. Furthermore, **CBK261309C** takes place in an allosteric binding site which induces significant structural distortions and prevents proper binding of NADPH. A complementary study of protein stability with the FabG enzyme of six pathogens in the presence of **CBK261309C** supports this mechanism, since lower melting points, due to decreased protein stability, were observed.

#### 2.4. ACP Dehydratases

##### 2.4.1. Description of FabA and FabZ

As mentioned in the introduction, FabA (β-hydroxydecanoyl-ACP dehydratase) and FabZ (β-hydroxyacyl-ACP dehydratase) catalyse the dehydration of β-hydroxyacyl-ACP in the third step of the elongation cycle [101,102,103,104,105]. FabA also performs isomerisation of *trans*-2- to *cis*-3-decenoyl-ACP as an essential step in the formation of unsaturated fatty acids, while FabZ only catalyses the dehydration reaction [48,103,104]. In addition, FabA is exclusively found in Gram-negative bacteria with its partner FabB, whereas FabZ is ubiquitously expressed in FAS-II systems [103,104,106]. FabA and FabZ do not share the same substrate selectivity, with C8-C12 and C6 substrates being recognized by FabA and FabZ, respectively [48,101]. The dehydratases involved in mycobacteria are called HadAB and HadBC. HadAB synthesizes mycolic acids with C46-C56 substrates, while HadBC forms longer mycolic chains (C58-C68) [107].

**Figure 20:**
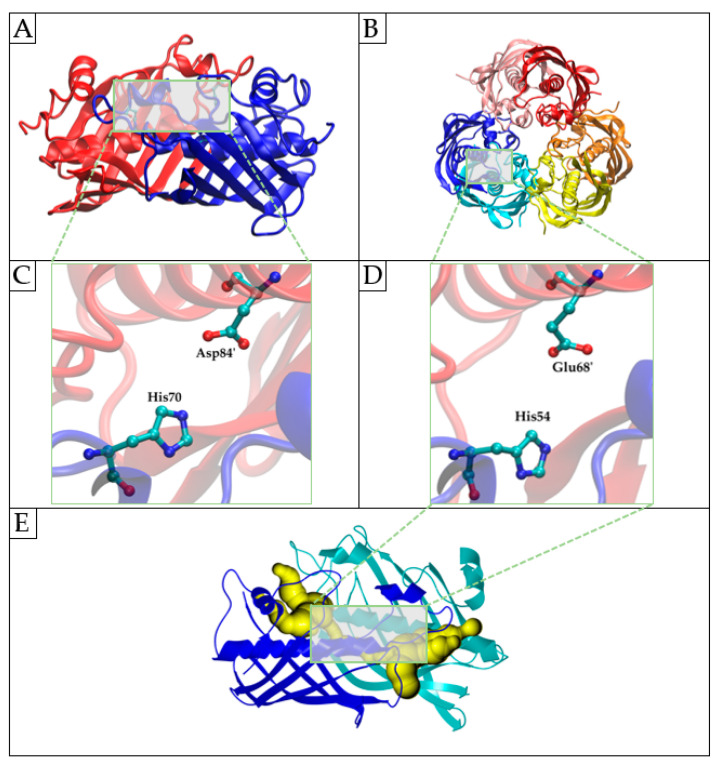
(**A**) *Ec*FabA apo-dimer (PDB ID 1MKB) structure. One FabA monomer is coloured in blue, the other in red. (**B**) *Ec*FabZ trimer of dimers (PDB ID 6N3P) structure. In each dimer, FabZ monomers are coloured in (i) cyan and blue, (ii) pink and red and (iii) orange and yellow. For clarity, the ACPs present in the structure are not shown. (**C**) View of the active site of *Ec*FabA (PDB ID 1MKB). This view is focused on the two catalytic residues Asp84′ and His70. (**D**) View of the active site of *Ec*FabZ (PDB ID 6N3P). This view is focused on the two catalytic residues Glu68′ and His54. The crosslinker present in the crystallographic structure was removed for clarity. (**E**) Tunnel topology of *Ec*FabZ dimer (PDB ID 6N3P). CAVER analyst was used to calculate the tunnel volume indicated as yellow surface. One FabZ monomer is coloured in blue, the other in cyan.

FabA structures from *E. coli* [108], *P. aeruginosa* [109] and *Yersinia pestis* [106] have been solved. Several FabZ structures have been reported in *E. coli* [101], *P. aeruginosa* [110], *P. falciparum* [111], *Francisella tularensis* [103], *Y. pestis* [103] and *Helicobacter pylori* [106], for instance. FabA and FabZ have highly related primary sequences. These homodimers adopt a β+α “hot dog” fold (Figure 20A,B). In solution, FabZ forms hexamers consisting of trimers of FabA-like dimers [101]. Their active site residues belong to both monomers and differ between FabA and FabZ, with His-Asn in FabA (Figure 20C) and His-Glu in FabZ (Figure 20D) [45,48]. The active sites of FabA and FabZ both lie within a tunnel, located alongside the central α-helix of each monomer (Figure 20E). Structures of *Ec*FabA are available in the PDB either in apo-form (PDB ID 1MKB) or in complex with ACP (PDB ID 4KEH), whereas the ACP-*Ec*FabZ complex (PDB ID 6N3P) is the only one available [101,108,112]. Interactions between ACP and the enzymes highlighted in these structures show that, in *Ec*FabA, His70 is involved in deprotonation and Asp84′ ensures correct substrate position (His54 and Glu68′ in *Ec*FabZ).

**Figure 21:**
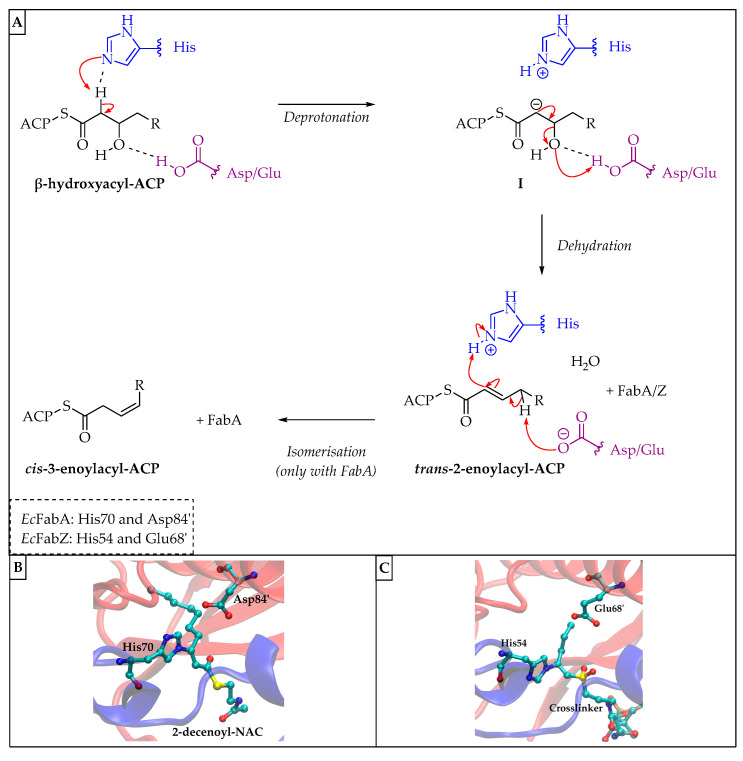
(**A**) Scheme of the mechanism of the reactions catalysed by *Ec*FabA and *Ec*FabZ. (**B**) View of the active site of *Ec*FabA (PDB ID 1MKA) in complex with 2-decenoyl-*N*-acetylcysteamine (NAC). The catalytic His70 is covalently bonded to 2-decenoyl-NAC. (**C**) View of the active site of *Ec*FabZ in complex with a cross-linker simulating intermediate **I** (PDB ID 6N3P).

Dehydration of β-hydroxyacyl-ACP occurs in two steps (Figure 21A). First, the hydrogen atom at C2 carbon of β-hydroxyacyl-ACP is transferred to His70 to afford **I** (Figure 21B). Then, the hydroxy group at C3 of intermediate **I** is protonated by Asp84′, and dehydration arises, yielding *trans*-2-enoylacyl-ACP. In *Ec*FabZ, this reaction is held by His54 and Glu63′ (Figure 21C). In the case of *Ec*FabA, a third step of isomerisation occurs in the same active site. Once the *trans*-2-unsaturated substrate is formed, the hydrogen atom is transferred from His70 back to C2 carbon, and the C4 carbon is deprotonated by Asp84′ to give the 3-*cis* product.

Due to its limited distribution in bacteria (only in Gram-negative), FabA does not seem interesting to develop broad-spectrum ATBs. [33]. Accordingly, FabZ appears to be the most attractive candidate for drug development in this group of enzymes [104].

##### 2.4.2. FabZ Inhibitors

**Figure 22:**
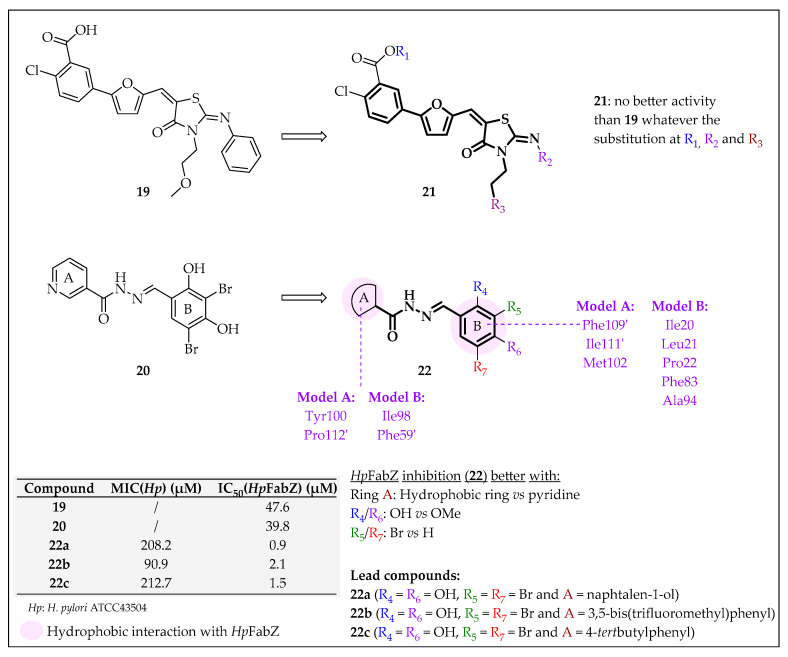
Structure, SAR and representation of main interactions with *Hp*FabZ of Schiff bases **19–22** and in vitro activities of lead compounds [102].

In 2008, Zhang et al. discovered two Schiff bases **19** and **20** (Figure 22) as *Hp*FabZ inhibitors (IC_50_ = 47.6 and 39.8 µM, respectively) using a surface plasmon resonance technology-based binding assay [106]. To improve their inhibitory activity, twenty-one analogues of **19** (**21**) and thirty-five derivatives of **20** (**22**) were synthesized (Figure 22) [102]. For **21**, the structural modification was mainly carried out by changing the imine substituent and the ether group (R_1–3_), while for **22**, the ring B was differently substituted (R_4–7_), and the nicotinohydrazide group was changed. Unfortunately, none of the few that were evaluated showed interesting ATB activity against *H. pylori*. The inhibitory activities of these compounds at 50 μM against HpFabZ were determined. However, for **21**, none of the compounds displayed better inhibitory activity than **19**. For **22**, as most of them displayed interesting inhibitory activity at 50 µM, their IC_50_ values were determined. The inhibitory activity of seven compounds **22** increased about four to twenty-eight times in comparison with that of compound **20**. SAR results indicated that the inhibitory activities decrease if (i) the bromine groups of the phenyl ring B are removed, (ii) the hydroxy substituent is replaced with methoxy, or (iii) the pyridine ring A is changed by phenyl ring, furan ring, methyl or 4-hydroxybenzyl. However, a more hydrophobic aromatic ring instead of pyridine, such as halogen or methoxy substituted phenyl or naphthalene, is favourable for the inhibition of *Hp*FabZ. Furthermore, the ATB potency of the best inhibitors (**22a–c**) was evaluated, but they displayed weak activity, with MIC values ranging from 90.9 to 212.7 µM. Docking studies with **22c**-*Hp*FabZ co-crystal resulted in two models. In model A, the inhibitor binds to the entrance of the *Hp*FabZ tunnel, while in model B, it is fixed to the middle of the tunnel near the active site. In model A, ring A of **22c** is sandwiched between Tyr100 and Pro112′, and ring B creates hydrophobic interactions with Phe109′, Ile111′ and Met102 which stabilize the inhibitor position. However, in model B, ring A interacts with Ile98 and Phe59′, while ring B is involved in several hydrophobic interactions with Ile20, Leu21, Pro22, Phe83 and Ala94 located almost at the end of the tunnel.

**Figure 23:**
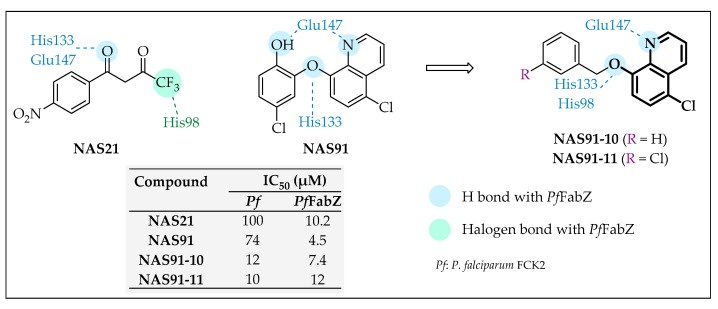
Structure, representation of main interactions with *Pf*FabZ and in vitro activities of **NAS** compounds [111,113].

In 2003, Sharma et al. tried to develop the first *Pf*FabZ inhibitors by designing aromatic compounds with a wide range of functional groups such as hydrazones, diketones or diaryl ethers [113]. However, these compounds displayed weak antiplasmodial activity (IC_50_(*Pf*) = 74 µM for the best compound **NAS91**). Among them, only **NAS21** and **NAS91** (Figure 23) inhibited *Pf*FabZ with IC_50_ values of 10.2 and 4.5 µM, respectively. To improve these activities, they synthesized **NAS91** analogues with methylene linker (**NAS91-10** and **NAS91-11**, Figure 23) [111]. These analogues exhibited similar inhibitory activity to those of **NAS91** but with an enhanced antiplasmodial potency. Moreover, a docking study brought to light two essential interactions created between these four inhibitors and His133 and Glu147 of *Pf*FabZ.

#### 2.5. Enoyl-ACP Reductases

##### 2.5.1. Description of FabI, FabK, FabL and FabV

As previously mentioned, reduction of the double bond in enoyl-ACP to acyl-ACP is catalysed by enoyl-ACP reductases (ENRs) in the ultimate and rate-limiting step of each elongation cycle round. ENRs comprise several enzymes: *trans*-2-enoyl-ACP reductase I (FabI), *trans*-2-enoyl-ACP reductase II (FabK), enoyl-ACP reductase III (FabL), and FabV [47,114]. ENRs are nicotinamide adenine dinucleotide (NADH)-dependent enzymes [115,116,117]. Among them, FabI is distributed broadly throughout most bacteria. FabI can be found alone, as in *S. aureus* and *E. coli*, or simultaneously with another ENR, as in *B. subtilis* with FabL or in *E. faecalis* with FabK [115,117,118]. FabK and FabV have been identified as the sole ENRs in *S. pneumoniae* and *Y. pestis*, respectively [17]. The FabI homologue in mycobacteria is termed InhA, and is essentially identical to the corresponding *E. coli* protein [50]. 

**Figure 24:**
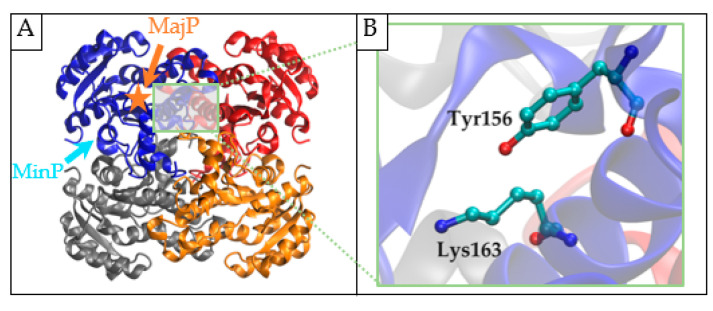
(**A**) *Ec*FabI apo structure (PDB ID 2FHS). The FabI monomers are coloured in blue, red, grey and orange. The AcpPs present in the structure were removed for clarity. The orange star symbolises the major portal (MajP), and the blue arrow represents the entry of the minor portal (MinP). (**B**) View of the active site of *Ec*FabI (PDB ID 2FHS). This view is focused on the two catalytic residues Tyr156 and Lys163.

Crystal structures of FabI (*E. coli* [119], *Bacillus anthracis* [120] and *P. aeruginosa* [114]), FabK (*E. coli* [117] and *S. pneumoniae* [121]), FabL (*B. subtilis* [122]) and FabV (*Y. pestis* [123]) are available in the PDB. In this part, only FabI will be described, since it is representative of all ENRs. FabI is a homotetramer in which each monomer adopts a characteristic Rossmann fold (Figure 24A) [119]. The active site of FabI is constituted by two conserved residues: Tyr and Lys (Figure 24B) [124]. Structures of EcFabI are available in complex with ACP (PDB ID 2FHS) [119] or with NAD^+^ (PDB ID 1DFI) [125]. Thanks to these structures, it was shown that in EcFabI, Lys163 secures the position of the cofactor, while Tyr156 is implicated in the reduction reaction. Two main entries to the active site pocket are available in ENRs: the minor and major portals (Figure 24A) [123,126].

**Figure 25:**
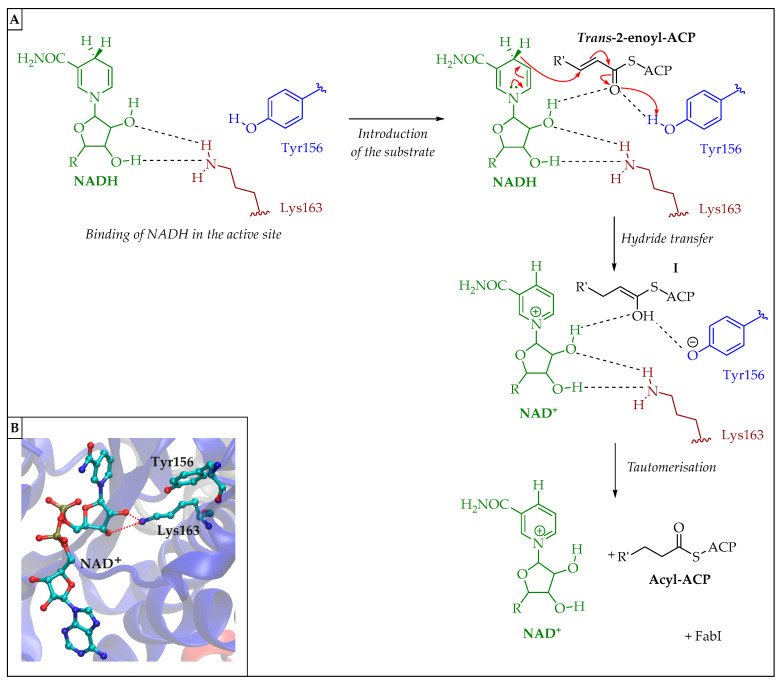
(**A**) Scheme of the mechanism of the reduction catalysed by *Ec*FabI. (**B**) View of the active site of *Ec*FabI-NAD^+^ structure (PDB ID 1DFI) highlights the hydrogen bonds between the hydroxy groups of NAD^+^ and Lys163 (red dots).

Reduction of *trans*-2-enoyl-ACP to acyl-ACP occurs in three main steps (Figure 25). First, the cofactor NADH forms hydrogen bonds with Lys163 to bind to the active site. Then, hydride transfer from NADH to the C3 of *trans*-2-enoyl-ACP occurs to obtain the enol **I**. After tautomerization of the intermediate **I**, acyl-ACP is synthesized [124]. The fatty acyl substrate and NAD^+^ binding site lies within a pocket composed of the major and minor portals.

Clinical success, mentioned in the introduction, has validated FabI as one of the most attractive enzymes of the FAS-II pathway. However, a specific FabI inhibitor has a relatively narrow spectrum of ATM activity, whereas an inhibitor targeting multiple ENRs should have broader-spectrum activity [117,127,128].

##### 2.5.2. FabI, FabK and FabL Inhibitors

###### Triclosan and Coumarin Derivatives

**Figure 26:**
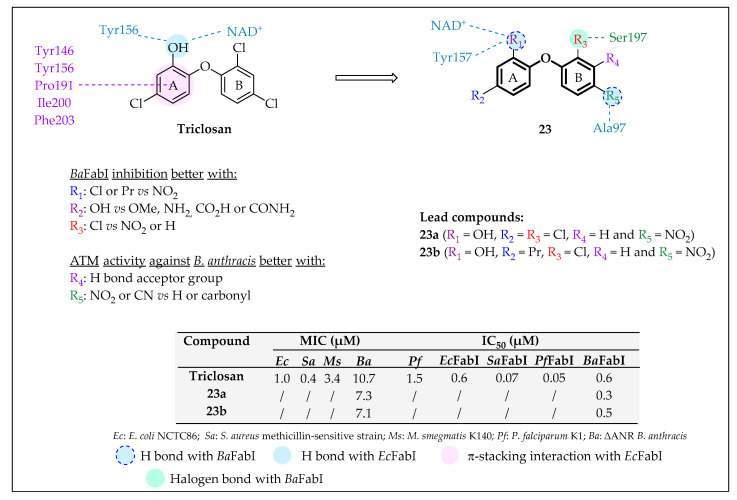
Structure, SAR and representation of main interactions with *Ec*FabI and *Ba*FabI of **triclosan** and its analogues **23** and in vitro activities of lead compounds [131,132,133,134,144,145].

**Triclosan** (Figure 26) has been used since the 1970s as a topical ATM in cosmetics, hygiene products and food [129]. This broad-spectrum ATM is active against Gram-negative [130] and -positive [117] bacteria, mycobacteria [131] and *P. falciparum* [132]. FabI was identified as one **triclosan** target in 1998 by McMurry et al. [8]. Moreover, it was showed that **triclosan** acts as a reversible inhibitor of FabI [133,134] and complexes with NAD^+^. It inhibits FabI of several pathogens such as *S. aureus*, *E. coli* and *P. falciparum* [117,130,132]. Docking studies with *Ec*FabI have shown that the hydroxy group of **triclosan** creates two hydrogen bonds with Tyr156 (part of the active site) and NAD^+^, simulating the intermediate **I** in the mechanism of the reduction by *Ec*FabI (Figure 25) [133,135]. Furthermore, van der Waals interactions are involved between the phenol ring of **triclosan** and Tyr146, Tyr156, Pro191, Ile200 and Phe203. While it is still legalised in the European Union with very strict regulations, the Food and Drug Administration took it off the American market in 2016 because of the potential health risks related to its long-term exposure [136]. The review of Weatherly et al., published in 2018, relates extensive information on **triclosan** impacts in human health [137]. For instance, it displays hormonal effects such as (i) an impact on the thyroid hormone homeostasis disrupting iodide uptake through sodium/iodide symporter modulation [138] and (ii) activation of pregnane X receptor-mediated transcription involved in steroid metabolism [139]. **Triclosan** exhibits mitochondrial toxicity that can induce long-term undesirable effects on somatic, reproductive, nervous and hepatic cells [140,141,142]. More precisely, **triclosan** seems to interfere with mitochondrial respiration through both a protonophoric effect and inhibition of complex II activity leading to apoptotic cell death [141]. More recently, Belosludtsev et al. hypothesised that **triclosan** can also induce mitochondrial toxicity by membranotropic effects (permeabilization of the plasmatic membrane, production of reactive oxygen species, influx of Ca^2+^) [143]. Thus, the synthesis of **triclosan** analogues with less adverse effects motivated medicinal chemists.

Tipparaju et al. worked on fifty-one **triclosan**-like aryl ether analogues **23** (Figure 26) to inhibit *Ba*FabI [144]. To highlight SAR, substituents of both phenyl rings were modulated. While the hydroxy group (R_1_) of ring A is critical for the inhibitory activity, (i) hydrogen bond donor at R_1_ and R_2_ and (ii) chloride atom at R_3_ of ring B result in better *Ba*FabI inhibitory activity. Furthermore, the ATM activity was increased with hydrogen bond acceptor groups at the 3- and 4-position of ring B (R_4_ and R_5_). The lead compounds **23a-b** displayed slightly better ATM activities against *B. anthracis* and inhibitory activity against *Ba*FabI than **triclosan**. It could be explained by the supplementary hydrogen bond created between the nitro group and Ala97. Docking studies with *Ba*FabI showed also that a halogen bond is formed between the chloro atom at position **2** (R_3_) and Ser197. The interactions created between the hydroxy group of **triclosan** and *Ec*FabI are still present between the hydroxy group in R_1_, NAD^+^ and Tyr157 (equivalent to Tyr156 in *Ec*FabI).

**Figure 27:**
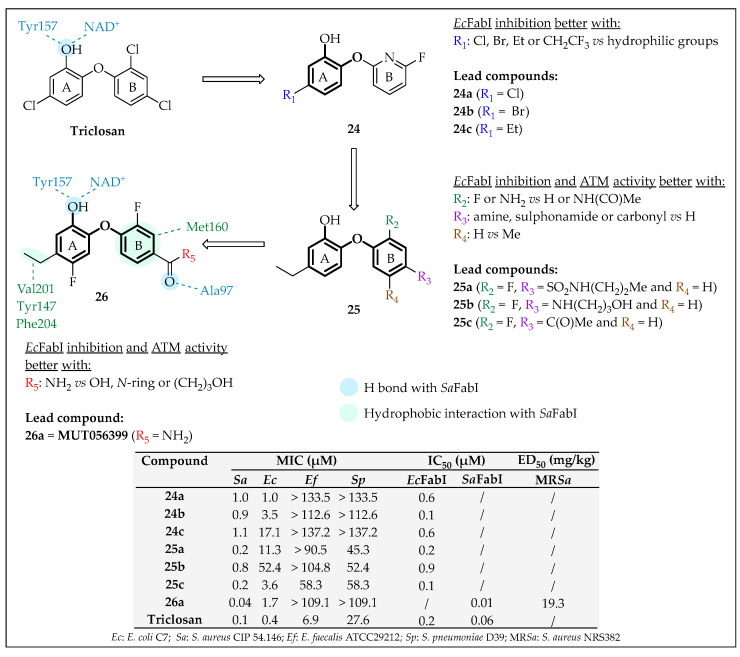
Structure, SAR and representation of the main interactions with *Sa*FabI of **triclosan** derivatives **24–26** and in vitro activities of lead compounds [146].

In 2012, Gerusz et al. engaged in rational drug-design study based on **triclosan** [146]. Thirty-two derivatives **24–26** (Figure 27) were designed by modulating substituents of both rings A and B and sometimes replacing the phenyl ring B by pyridine (**24**). In the first instance, fifteen derivatives **24** were designed by changing the nature of the substituents in R_1_ of the ring A, while ring B was a 2-fluoropyridine. Replacing the chloride (**24a**) by a bromide led to improved *Ec*FabI inhibition (IC_50_ = 0.1 vs. 0.6 µM), while alkyl and fluoroalkyl groups were well tolerated. However, no compound displayed better ATB activity than **24a** against *E. coli*. Modulation of the ring B substituents of **25** highlighted that (i) fluoride in *ortho*-position (R_2_) increases both inhibitory and ATM activities, (ii) *para-*substitution (R_3_) with sulphonamides, amines or carbonyls is well tolerated, and (iii) *meta*-substitution (R_4_) strongly decreases the inhibitory activity. Furthermore, compounds **25a-c** displayed dual activity, since they also inhibited growth of *S. pneumoniae*, which possesses exclusively FabK as ENR. Both broad-spectrum activity and the strong antistaphylococcal activity of **25c** (MIC = 0.2 µM) led to its pharmacomodulation. While fluoride was added on the ring A, the *para*-acetyl group was substituted with more hydrophilic derivatives (amides or carboxylic acids). Among the seven compounds **26** synthesized, all displayed interesting inhibitory activity (IC_50_(*Sa*FabI) = 0.01-0.2 µM). Nonetheless, **26a** exhibited better antistaphylococcal activity than **triclosan** (MIC = 0.04 vs. 0.1 µM) and no cytotoxicity on HepG2 cell lines. A docking study with this promising compound, also called **MUT056399**, and *Sa*FabI highlighted: (i) hydrophobic interactions between the ethylic chain of **26a** and the triad Val201-Tyr147-Phe204, (ii) hydrogen bonds between ring A, Tyr157 and NAD^+^ (as observed with *Ec*FabI and **triclosan**, Figure 26), (iii) hydrophobic interactions between ring B and Met160 and (iv) hydrogen bond between the ring B oxygen carbonyl and Ala97. Preclinical studies of **26a** on mice and dogs did not exhibit toxicity or safety risks which could prevent its use. Furthermore, the in vivo efficacy on mice validated **26a**, as an interesting antistaphylococcal, with ED_50_ values ranging between 19.3–45.1 mg/kg for several methicillin-resistant *S. aureus* strains. However, the preclinical assays seem to be stopped for unknown reasons.

**Figure 28:**
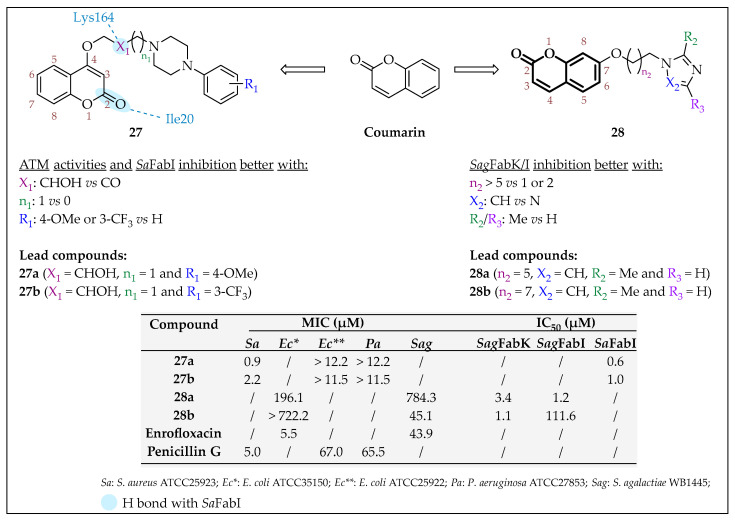
Structure, SAR and representation of main interactions with *Sa*FabI of coumarin derivatives **27–28** and in vitro activities of lead compounds [147,148].

In 2014, Wang et al. worked on coumarin derivatives **27** (Figure 28) to develop *Sa*FabI inhibitors [147]. During SAR study, a substituted phenylpiperazine was introduced at the position 4 of coumarin through the aliphatic chain including an alcohol function or carbonyl group. It was noticed that a linker with (i) hydroxy group (X_1_) instead of ketone, (ii) four-unit length (n = 1) and (iii) *para*-substituted phenyl (R_1_), especially with methoxy group, encouraged both inhibitory and ATM activities. Unfortunately, while derivatives **27** were generally more active against Gram-positive bacteria than the reference penicillin G, they did not inhibit Gram-negative bacteria. Docking studies between the lead compound **27a** and *Sa*FabI revealed that two hydrogen bonds are created between (i) Lys164 and the hydroxy group of **27a** and (ii) Ile20 and the carbonyl oxygen of **27a**.

In 2018, Hu et al. designed thirty-nine other coumarin derivatives **28** with an imidazole rink linked by an alkyloxy arm at C7 position (Figure 28) as *Streptococcus agalactiae* FabK (*Sag*FabK) inhibitors [148]. The length of the carbon-chain linker was modulated, and the imidazole ring was substituted with methyl or phenyl groups (R_2–3_). The SAR study showed that at least six carbons (n = 5) in the chain were necessary to inhibit *Sag*FabK. In addition, they noticed that methylation in position 2 or 4 of the imidazole increased the inhibitory potential of compounds. Moreover, there was a strong correlation between ATM activities and anti-FabK activity. Generally, these compounds were poor FabI inhibitors except for **28a** (IC_50_(*Sag*FabI) = 1.2 µM). Surprisingly, two additional carbons in the aliphatic chain decreased the affinity for *Sag*FabI but maintained it for *Sag*FabK.

**Figure 29:**
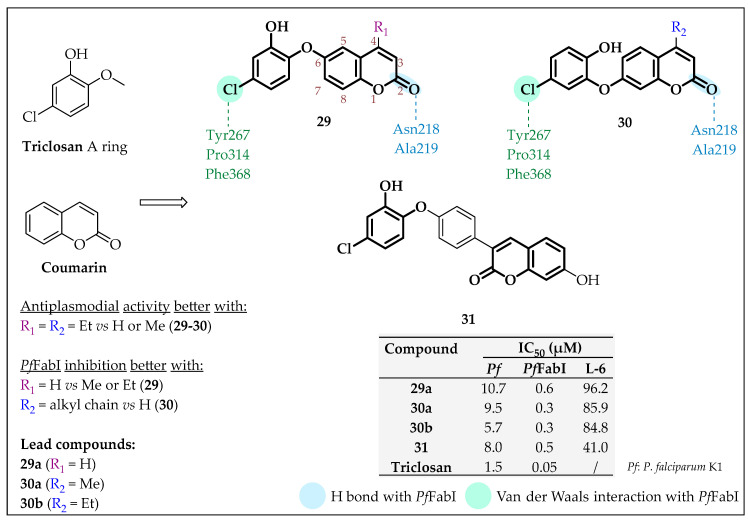
Structure, SAR and representation of main interactions with *Pf*FabI of **triclosan** derivatives **29–31** and in vitro activities of lead compounds [132].

In 2013, Belluti et al. coupled triclosan and coumarin to develop *Pf*FabI inhibitors [132]. Twelve compounds divided into two series of hybrids with the **triclosan** A ring inserted at position 6 (**29**, Figure 29) or position 7 (**30**, Figure 29) of coumarin and a thirteenth compound (**31**, Figure 29) with a phenyl linker between both units were designed. Interestingly, in compounds **29**, alkyl chain substituents (R_1_) at position 4 of the coumarin hybrid decreased inhibitory activity while improving it in compounds **30** (R_2_). Consequently, in the first series, the lead compound **29a** was not substituted (R_1_ = H), while the lead compounds of the second series **30a–b** were methyl- or ethyl-substituted (R_2_ = Me or Et). However, in both series, compounds with the best antiplasmodial activity were substituted with ethyl at the position 4. Compound **31** (IC_50_(*Pf*FabI) = 0.5 µM) was less cytotoxic than the others on mammalian L-6 cells. Docking studies showed that the **triclosan** A ring and coumarin ring of compounds **30** and **31** interact in the same way with *Pf*FabI. Indeed, the 5-chloro of **triclosan** A ring induced van der Waals interactions with Tyr267, Pro314 and Phe368. The coumarin-2-one group formed hydrogen bonds with Asn218 and Ala219.

###### Pyridine, Pyridone and Pyrone Derivatives

**Figure 30:**
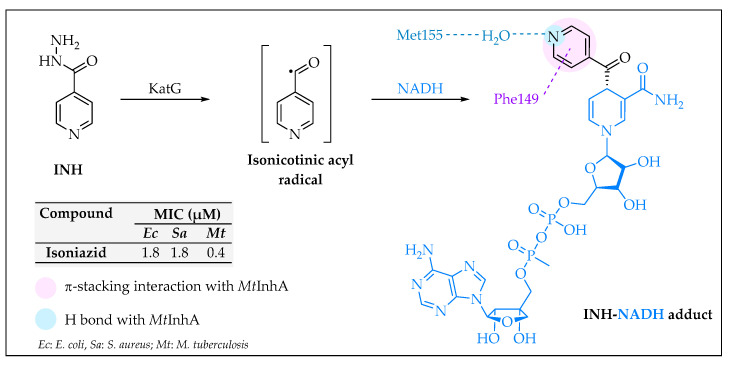
Structure, representation of the main interactions with *Mt*InhA and in vitro activities of **INH** and its active form bound to NADH [7,47,126].

**Isoniazid** (**INH**, Figure 30) was introduced in 1952 into the antituberculosis arsenal and is still used in first-line treatment in combination with pyrazinamide, rifampicin and ethambutol [149,150]. However, **INH** also displayed ATB activity against *E. coli* and *S. aureus* with MIC of 1.8 µM [111]. In 1995, Quemard et al. identified InhA (the analogue of FabI in mycobacteria) as its target [7]. **INH** is a prodrug that has to be activated by KatG catalase-peroxidase into isonicotinic acyl radical [151,152]. Rozwarski et al. have shown that the isonicotinic acyl group binds covalently to NADH cofactor at the C4 of its nicotinamide ring and replaces the 4*S* hydrogen atom necessary in the hydride transfer of the reductase reaction (Figure 25) [126]. The resulting **INH**-NADH complex lies within InhA and induces its conformational rearrangement, allowing two main interactions: (i) the π-stacking interaction between Phe149 and the pyridine moiety of the adduct and (ii) a water-mediated hydrogen bond created between the pyridinic nitrogen of **INH**-NADH adduct and Met155 of MtInhA. Nevertheless, even if the ATB activity of **INH** should be accountable for its interaction with FabI, no data certified it. However, **INH** displays severe side-effects on hepatocytes and the central nervous system. Hepatotoxicity is partly induced by hepatic accumulation of protoporphyrin IX through distortion of heme biosynthesis [153], while neurotoxicity can be explained by (i) a defective neurotransmission (reduction of glutamate and gamma-aminobutyric acid levels) and (ii) induction of osmotic stress [154].

**Figure 31:**
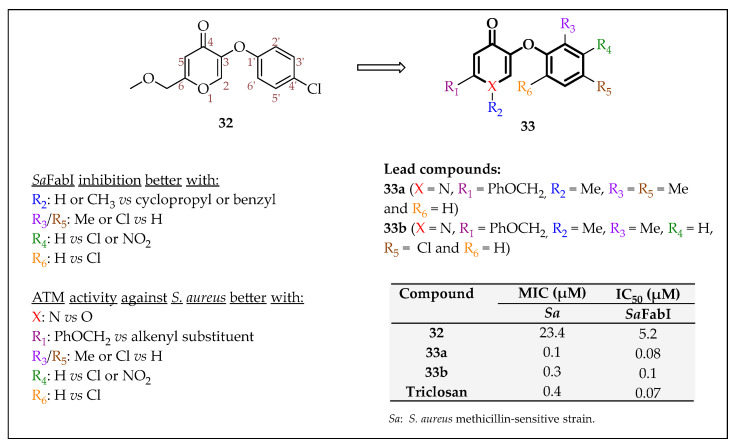
Structure and SAR of **32–33** and in vitro activities of lead compounds [145].

Thanks to a high-throughput screening, Kim et al. identified phenoxypyrone **32** (Figure 31) as an interesting *Sa*FabI inhibitor (IC_50_ = 5.2 µM) [145]. To increase its inhibitory and ATM activities, fifty-one derivatives **33** (Figure 31) were developed by modulating the substituents of both rings. To improve the solubility and the physico-chemical properties of their compounds, 4-pyrone ring was replaced by 4-pyridone. SAR study showed that 4-pyridone with (i) phenoxymethyl substituents at position 6 (R_1_) lowered the MIC against *S. aureus*, and (ii) bulky substituents or (iii) long carbon-chain at position 1 (R_2_) decreased the inhibitory activity. Furthermore, 2′,4′-disubstituted phenoxy (R_3_ and R_5_) at position 3 of 4-pyridone led to compounds with better inhibitory and ATM activities than 2′,3′- (R_3_ and R_4_) or 2′,6′-disubstituted (R_3_ and R_6_), whether the substituents were EW or ED groups. Based on these SAR, the lead compounds **33a** and **33b** exhibited better ATM and inhibitory activity (IC_50_(*Sa*FabI = 0.08–0.1 µM) than **32**.

**Figure 32:**
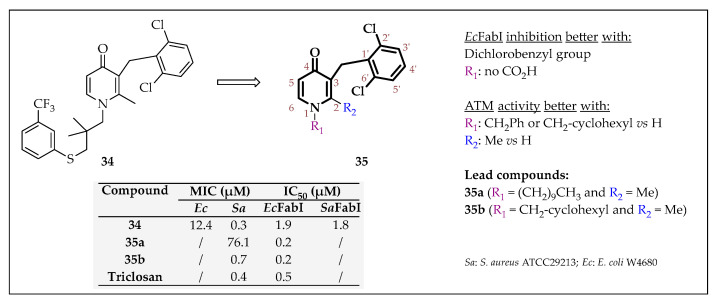
Structure and SAR of 4-pyridone derivatives **34** and **35** and in vitro activities [155,156].

In 2007, Kitagawa et al. identified 4-pyridone **34** (Figure 32) as an *Ec*FabI and *Sa*FabI inhibitor (IC_50_ = 1.9 and 1.8 µM, respectively) through high-throughput screening [155,156]. SAR analysis of thirty derivatives **35** was realised by modulating the *N*-substituent at R_1_ and the length of the alkyl chain on R_2_. It was showed that the 2′,6′-dichlorobenzyl group was necessary to inhibit *Ec*FabI. Furthermore, substituted pyridones (R_1_) were generally more active than the unsubstituted, except when the substituent carried carboxylic acid. Higher ATM activities were observed with hydrophobic groups at R_2_, such as either a saturated or unsaturated cyclic group or alkyl chain. This SAR study led to two lead compounds **35a** and **35b**, which displayed better inhibitory activity (IC_50_(*Ec*FabI) = 0.2 µM for both) than **34**.

**Figure 33:**
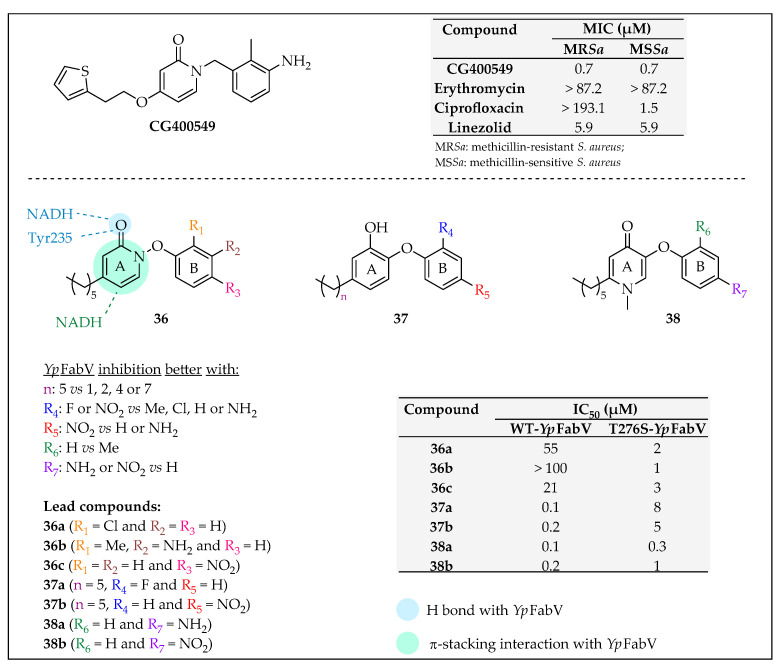
Structure, SAR and representation of main interactions with *Yp*FabV of **CG400549** and **36–38** and in vitro activities of lead compounds [17,157].

In 2007, a team of CrystalGenomics described one 2-pyridone, **CG400549** (Figure 33), with strong in vitro and in vivo antistaphylococcal properties [157]. Indeed, it was the most active against sixty-nine methicillin-sensitive and one hundred and sixty-nine -resistant clinical strains (MIC = 0.7 µM) compared to the references erythromycin, ciprofloxacin or linezolid. *Sa*FabI was indirectly highlighted as the target of **CG400549**: (i) the MIC of the pyridine was sixty-four folds higher on a FabI-overexpressing strain compared to parental strain, and (ii) a mutation in FabI at Phe204 to Leu was identified as the prime reason for resistance in *S. aureus* **CG400549**-resistant strains. Furthermore, in vivo study in mice showed that this compound was active when administered both orally (ED_50_ = 4.4 mg/kg) or by subcutaneous route (ED_50_ = 18.9 mg/kg). Based on these encouraging data, CrystalGenomics started in 2012 a phase 2a clinical trial of **CG400549** in the treatment of a small cohort infected by methicillin-resistant *S. aureus* (NCT01593761). The analysis of the results of this study has not been published yet.

In 2012, Hirschbeck et al. described the structural analysis of other 2-pyridones **36a** and **36b** (Figure 33) in complex with *Yp*FabV-NADH and highlighted that (i) the carbonyl oxygens of both **36a** and **36b** bind with Tyr235 and NADH via hydrogen bonds, and (ii) ring A forms π-stacking interactions with the nicotinamide ring of NADH [123]. Furthermore, this analysis showed some mechanistic differences between the functioning of typical FabI isoforms and *Yp*FabV. Indeed, *Yp*FabV contains additional residues that are mainly located around the substrate-binding loop. This loop plays an essential role in FabI since it adapts to the size of ligands and is closed when ligands are bound. In *Yp*FabV, it follows an inverse mechanism with a closed conformation in *apo* form and opens to enable access to the natural substrate and potential inhibitors. Additionally, Thr276 (located at the *N*-terminus of the substrate-binding loop) was identified, during cloning, as a key residue to explore. Later, the same team described the characterisation of wild-type (WT) *Yp*FabV and Tyr276 mutants [17]. These mutants displayed similar catalytic efficiencies, but most of them possessed reduced activities compared to WT-*Yp*FabV. Additionally, structural analysis of WT-*Yp*FabV and its T276 mutants revealed that the substrate-binding loop adopts a closed conformation for both WT- and T276S-*Yp*FabV, whereas in the other mutants this loop is in a more open conformation inducing a loss of stability. In addition, a structure-based drug design study was carried out using 2-pyridones **36**, diphenylethers **37** and 4-pyridones **38** (Figure 33) as scaffolds and WT-*Yp*FabV and T276S-*Yp*FabV as enzymes. For diphenylethers **37**, structural variations were carried out (i) on ring A by modulating the length of the carbon chain and (ii) on ring B by changing the nature and the position of the substituents. SAR study revealed that diphenyl ethers **37** non-substituted on ring B prefer propyl substituent (n = 2) on ring A instead of shorter (n = 1) or longer (n = 7) alkyl substituents. However, *ortho*-fluorine (R_4_) or *para*-nitro (R_5_) groups are helpful for *Yp*FabV (WT and T276S mutant) inhibition when ring A is substituted by an *n*-hexyl group. In both 2-pyridones **36** and 4-pyridones **38**, the *n*-hexyl chain was fixed, while the ring B was modulated, using methyl, amino or nitro groups. *Ortho*-methyl groups are detrimental to inhibitory activities but *para*-nitro and -amino groups are well tolerated. These led to four lead compounds **37a-b** and **38a-b** with interesting inhibitory activities against WT-*Yp*FabV (IC_50_ = 0.1–0.2 µM). While 2-pyridones **36** targeted preferentially T276S-*Yp*FabV, and diphenylethers **37** the WT enzyme, 4-pyridones **38** displayed similar potency against both WT- and T276S-*Yp*FabV. Hence, the activity of 4-pyridones **38** is less dependent on the active site architecture, suggesting broad-spectrum activity.

###### Imidazole Derivatives

**Figure 34:**
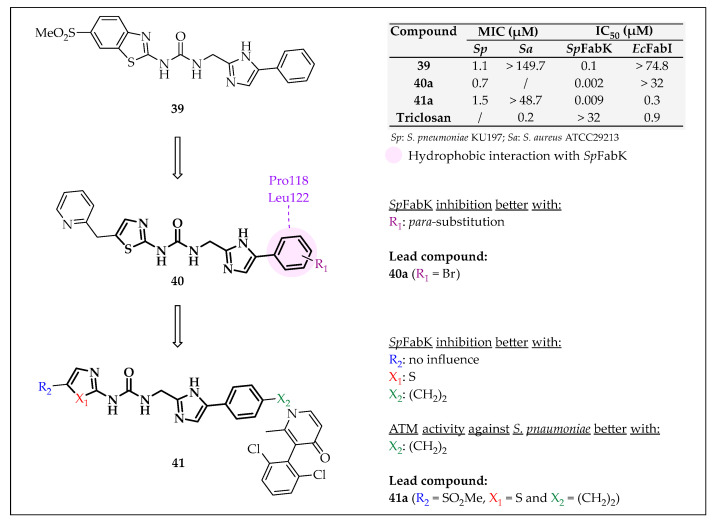
Structure, SAR and representation of main interactions with *Sp*FabK of phenylimidazole derivatives **39–41** and in vitro activities of lead compounds [158].

Kitagawa et al. identified **39** (Figure 34) as *Sp*FabK inhibitor (IC_50_ = 0.1 µM), which correlates with its whole-cell activity (MIC = 1.1 µM against *S. pneumoniae*) [158]. However, it did not inhibit *Ec*FabI or *S. aureus* growth. To broaden the spectrum of activity of **39** toward other ENRs, four phenylimidazole derivatives **40** were developed, but none of them inhibited *Ec*FabI. Nonetheless, docking studies with *Sp*FabK and the lead compound **40a** (IC_50_(*Sp*FabK) = 0.002 µM) showed that Pro118 and Leu122 create hydrophobic interactions with the phenyl ring. Based on these observations, they noticed that *para*-substituted phenyl (R_1_) improved *Sp*FabK inhibition. Hence, nine derivatives **41** were designed by substituting R_1_ with 4-pyridone derivatives, FabI inhibitor scaffolds already described by this team (compounds **34** and **35**). The following SAR were highlighted: (i) thiazole group (X_1_ = S) and (ii) two-carbon alkyl chain (X_2_), as linkers between the 4-pyridone moiety and the phenylimidazole, were favourable to *Sp*FabK inhibition. This led to the lead compound **41a,** which inhibited both *Sp*FabK and *Ec*FabI (IC_50_ = 0.009 and 0.3 µM, respectively) and displayed good ATM activity against *S. pneumoniae* (MIC = 1.5 µM). Nevertheless, none of the synthesized derivatives exhibited ATM activity against either *E. coli* or *S. aureus*.

**Figure 35:**
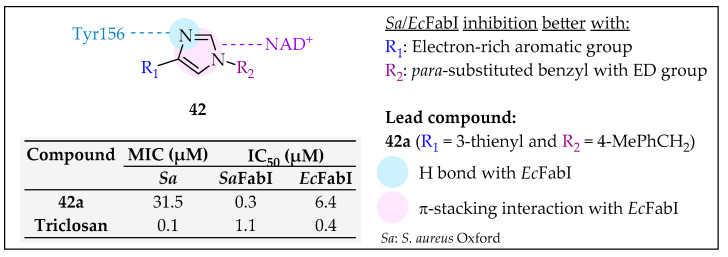
Structure, SAR and representation of main interactions with *Ec*FabI of imidazoles **42** and in vitro activities of lead compound [159].

In 2001, a team of GlaxoSmithKline Pharmaceuticals used high-throughput screening to discover *Sa*FabI inhibitors. They identified 1,4-disubstituted imidazoles **42** (Figure 35) [159]. Heerding et al. demonstrated that imidazole substituted with electron-rich groups were well tolerated at the 1- and 4-positions (R_1_ and R_2_). Moreover, a benzyl ring with a small ED group at the *para*-position at R_2_ displayed better *Sa*FabI and *Ec*FabI inhibitory activity. The lead compound **42a** exhibited better inhibition against *Sa*FabI than **triclosan** (IC_50_ = 0.3 vs. 1.1 µM) but was less effective against *Ec*FabI and *S. aureus*. A docking study with **42a** and *Ec*FabI showed that (i) the unsubstituted imidazole nitrogen forms a hydrogen bond with Tyr156, member of the active site, and (ii) the thiophene ring is engaged in a π-stacking interaction with the nicotinamide ring of NAD^+^.

###### From Tetrahydro-1,4-benzodiazepine Derivatives to Afabicin

**Figure 36:**
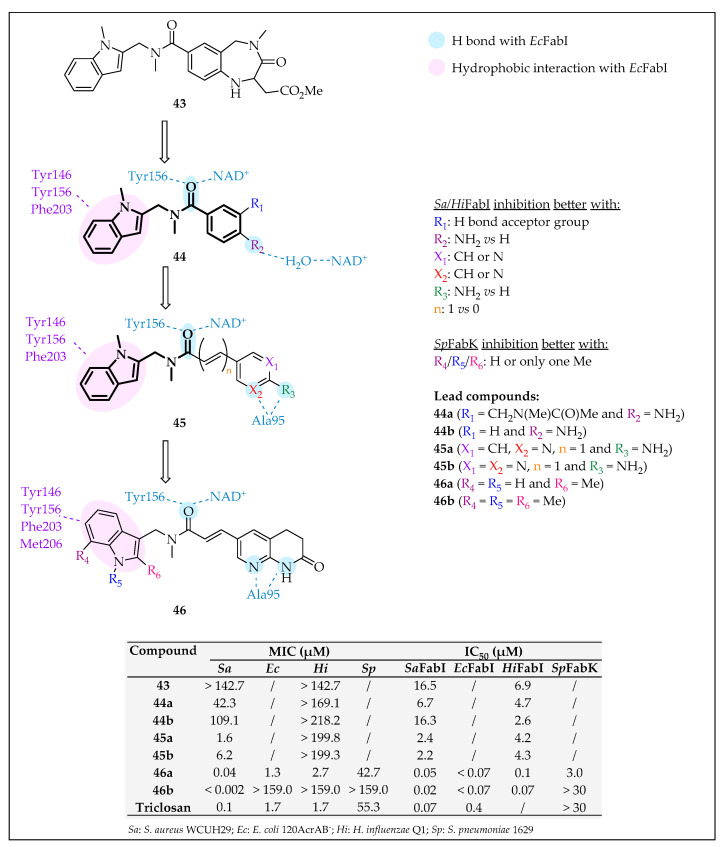
Structure, SAR and representation of main interactions with *Ec*FabI of **43–46** and in vitro activities of lead compounds [128].

A high-throughput screening conducted by GlaxoSmithKline Pharmaceuticals allowed the discovery of 1,4-benzodiazepine **43** (Figure 36) as a *Sa*FabI inhibitor [128]. Unfortunately, **43** did not exhibit activity against *S. aureus* and *Haemophilus influenzae*. However, this compound was slightly active against *Sa*FabI and *Hi*FabI (IC_50_ values of 16.5 and 6.9 µM, respectively). First, SAR analysis had already revealed the importance of the indole group, which was conserved by focusing on the modification of the benzodiazepine subunit. Particularly, they explored the effect of simplified ring-opened analogues **44**. As expected, *para*-aminobenzamides **44a** and **44b** were more active than **43** against both *Sa*FabI and *Hi*FabI enzymes (IC_50_ against *Sa*/*Hi*FabI of 6.7/4.7 and 16.3/2.6 µM for **44a** and **44b**, respectively), additionally displaying antistaphylococcal activity (MIC = 42.3 µM). With the minimum pharmacophore thus highlighted, a structural analysis of the **44a**-*Ec*FabI X-ray co-crystal structure in the presence of NAD^+^ was carried out. Several key interactions were brought to light: (i) the indole group binds with Tyr146, Tyr156 and Phe203 (located in a lipophilic enzymatic pocket) through hydrophilic interactions, (ii) the carbonyl oxygen of the amide creates two hydrogen bonds with Tyr156 and NAD^+^, and (iii) the primary amine interacts with NAD^+^ through a water molecule and seems to simulate the enol intermediate **I** (Figure 25). With this consideration as well as previous study in mind, Miller et al. replaced the aniline subunit by an aminopyridine moiety bound to the amide group through either an ethyl or ethenyl linker. Compounds **45a-b** were the most active analogues in both enzymatic (IC_50_(*Sa*/*Hi*FabI) = 2.4/4.2 and 2.2/4.3 µM, correspondingly) and whole cell anti-*S. aureus* assays (MIC of 1.6 and 6.2 µM, respectively), surpassing the activity of **43** and **44a-b**. X-ray co-crystal structure analysis of **45a**-*Ec*FabI/NAD^+^ showed that **45a** binds in the active site with the same interactions previously described for **44a** concerning amide and indole groups. However, a different binding mode was highlighted for the aminopyridine group compared to the aniline moiety, since it creates two hydrogen bonds with Ala95. Further in vivo study carried out with **45a** showed that the inhibitor was effective in an *S. aureus* groin abscess infection model in rats at 50 mg/kg, reducing by 2.5 log relative to untreated controls the bacterial counts.

To continue this work, in 2003 another team of GlaxoSmithKline Pharmaceuticals designed napthypyridinyl-based FabI and FabK inhibitors **46** (Figure 36) [117]. They noticed that both indole ring and amide function are necessary to inhibit FabI. They showed that substitution of the indole ring is limited to small lipophilic groups. Moreover, the presence of more than one methyl group on this ring (R_4_, R_5_ or R_6_) induces loss of *Sp*FabK and *S. pneumoniae* inhibition. The lead compounds **46a** and **46b** both inhibited *Sa*FabI, *Hi*FabI, and *Ec*FabI. However, **46a** was the only one which possessed broad-spectrum ATM activity (MIC = 0.04–42.7 µM against *S. aureus*, *E. coli*, *H. influenzae* and *S. pneumoniae*) and targeted *Sp*FabK (IC_50_ = 3.0 µM). Docking studies with **46a** and *Ec*FabI highlighted the main interactions involved. Both nitrogen atoms of the naphthyridinone form hydrogen bonds with Ala95. Furthermore, the amide carbonyl appears to participate in hydrogen bonds with NAD^+^ and Tyr156 (part of the active site). Hydrophobic interactions are created with indole and the lipophilic enzymatic pocket constituted of Tyr146, Tyr156, Phe203 and Met206. The substitutions of indole with bulky groups disrupt these interactions.

**Figure 37:**
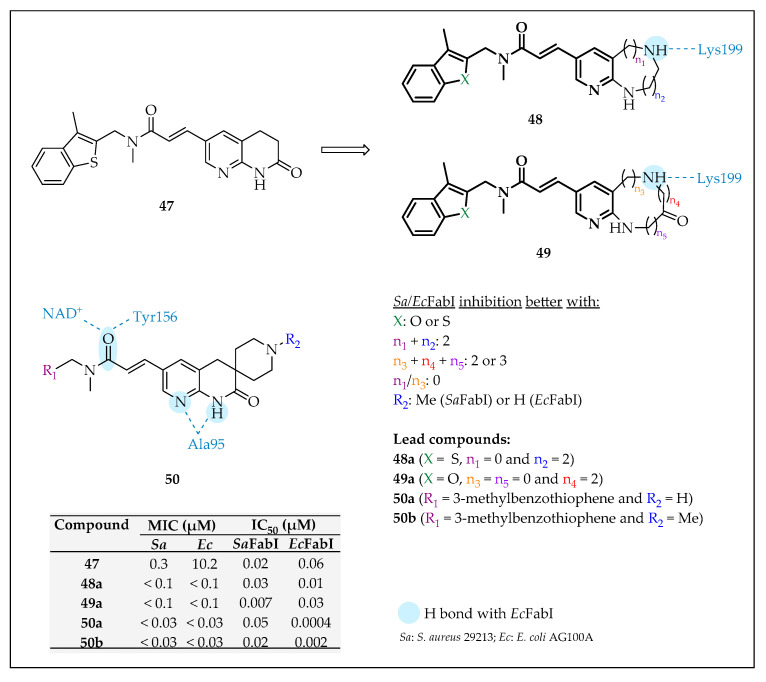
Structure, SAR and representation of main interactions with *Ec*FabI of **47–50** and in vitro activities of lead compounds [160,161].

Based on the work of GlaxoSmithKline Pharmaceuticals, Affinium Pharmaceuticals began to work on this family of compounds in 2009. Ramnauth et al. identified the naphtyridinylacrylamide **47** (Figure 37) as an *Sa*FabI and *Ec*FabI inhibitor [160]. To increase its ATM activity against *E. coli*, modulation of five diazepines (n_1_ + n_2_ = 1) or diazocines (n_1_ + n_2_ = 2) **48** and nine diazepinones (n_3_ + n_4_ + n_5_ = 2) or diazocinones (n_3_ + n_4_ + n_5_ = 3) **49** was carried out. Some SAR were identified: (i) diazepinones **49** were more potent *Sa/Ec*FabI inhibitors than diazepines **48**, (ii) seven- and eight-membered rings could be tolerated, and (iii) benzofurans (X = O) were more active than benzothiophenes or indoles (X = S or NH). The lead compounds **48a** and **49a** were active against *S. aureus* and *E. coli* efflux pump mutant and their respective FabI enzymes. A docking study with *Sa*FabI, showed that 1,5-diazepinones could be more effective against FabI than 1,4-diazepinones, probably due to a tighter hydrogen bond with Lys199 (*Sa*FabI).

Sampson et al. worked on the same family of compounds and designed analogues **50** (Figure 37) as *Sa*FabI inhibitors [161]. While the nature of R_1_ did not influence the potency, compounds with free amine instead of methylated amine (R_2_ = H or Me, respectively) displayed much better inhibitory activity against *Ec*FabI (IC_50_ = 0.0004 vs. 0.03 µM). The lead compounds **50a** and **50b** inhibited the growth of both *S. aureus* and *E. coli* (MIC lower than 0.03 µM) and exhibited good inhibitory activity against *Sa*FabI (IC_50_ = 0.05 and 0.02 µM, respectively) and *Ec*FabI (IC_50_ = 0.0004 and 0.002 µM, respectively). A docking study showed that the hydrogen bonds observed between **44–46** (Figure 36) and *Ec*FabI are also formed between **50** and the enzyme: (i) amide carbonyl interacts with Tyr156 and NAD^+^ and simulates intermediate **I** in the *Ec*FabI mechanism (Figure 25), and (ii) the pyridyl nitrogen and amide hydrogen of the naphthyridinone ring are involved in hydrogen bonds with Ala95.

**Figure 38:**
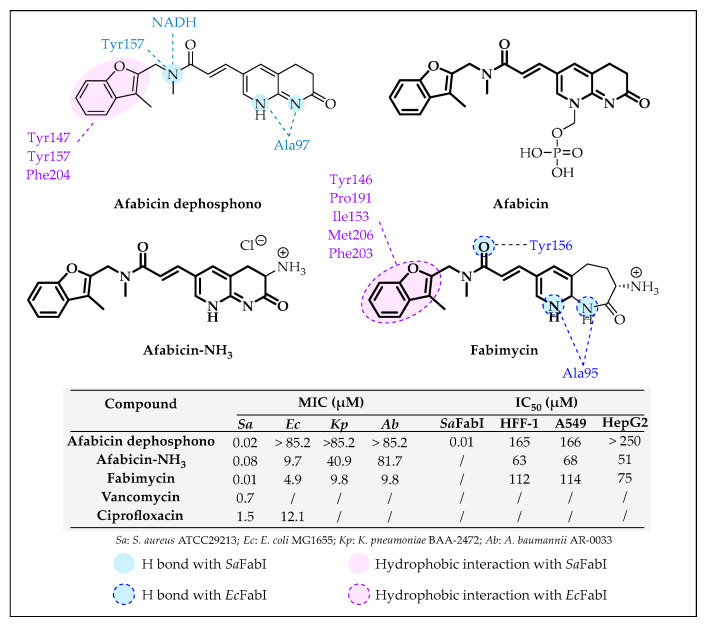
Structure, interactions with *Sa*FabI and *Ec*FabI and in vitro activity of **afabicin dephosphono** and its analogues [115,163,164].

These SAR studies led to the discovery of the sole inhibitor of one FAS-II enzyme currently in clinical development: **Afabicin dephosphono** (Figure 38), previously called API-1252, AFN-1252 or Debio-1452, which was discovered in 2007 by Affinium Pharmaceuticals [162]. Its structure is composed of 3-methylbenzofuran ring and an oxotetrahydronaphthyridine moiety linked by an *N-*methylpropenamide. This compound displays exceptional ATM activity against sensitive and resistant strains of *S. aureus* [163]. However, **afabicin dephosphono** displays poor ATM activity against Gram-negative bacteria because of efflux mechanisms and difficulties to penetrate bacteria membranes [163,164]. It targets specifically *Sa*FabI and very weakly affects the FAS-I system (FabI/FAS-I selectivity ratio > 4800) [115]. Banevicius et al. demonstrated that there is no concentration-dependent binding to FabI for **afabicin dephosphono** [165]. Docking studies with *Sa*FabI showed that the inhibitor impairs the formation of the intermediate I of the FabI mechanism (Figure 25). Indeed, the carbonyl of the *cis*-amide of **afabicin dephosphono** interacts with NADPH and Tyr157 (equivalent to Tyr156 in *Ec*FabI). Both the naphtypyridine nitrogens are involved in hydrogen bonds with Ala97. The furan ring is nestled in a lipophilic enzymatic pocket defined by Tyr147, Tyr157 and Phe204 [115]. **Afabicin-NH_3_** and the prodrug **afabicin** were synthesized to inhibit Gram-negative bacteria and to improve the drug-like properties, such as the hydrosolubility, respectively (Figure 38) [164]. **Afabicin-NH_3_** was designed using eNTRy way (presence of a primary amine, low globularity and few rotatable bonds), a strategy developed to broaden the spectrum of ATB effective against Gram-negative bacteria. As afabicin inhibited Gram-positive bacteria and was already conformed with two of the three eNTRy rules (low globularity and few rotatable bonds), it only lacked a primary amine to respect the entire conditions. As expected, **Afabicin-NH_3_** was active against both *S. aureus* and *E. coli.* Two phase II clinical trials were conducted: (i) in 2012, **afabicin dephosphono** was tested in the treatment of staphylococcal infections of the skin (NCT01519492), and (ii) afabicin is currently being assessed in the treatment of bone and joint infections due to *S. aureus* (NCT03723551) by Debiopharm International. To develop a more promising candidate for Gram-negative infections with better in vivo tolerance, Parker et al. developed **fabimycin** (Figure 38) based on **afabicin dephosphono** and afabicin-NH_3_ by extending and reducing the tetrahydronaphthyridinaminium to hexahydropyridoazepinaminium [166]. **Fabimycin** displayed broad-spectrum activity against Gram-positive (MIC = 0.01 µM against *S. aureus*) and -negative (MIC ranging 4.9–9.8 µM against *E. coli*, *K. pneumoniae* and *A. baumannii*) bacteria. Nevertheless, **fabimycin** is lethal only for bacteria that possess exclusively FabI as ENR. Cytotoxic evaluations against three human cell lines (HFF-1, A549 and HepG2) revealed that **fabimycin** was less cytotoxic than afabicin-NH_3_ but more cytotoxic than **afabicin dephosphono**. Additionally, it was nonhemolytic at 200 µM. In murine infection models using mice infected with drug-resistant *A. baumannii* or *S. aureus*, **fabimycin** was more efficient than afabicin-NH_3_. **Fabimycin**-*Ec*FabI co-crystal analysis highlighted several key interactions, equivalent to those within the **afabicin dephosphono**–*Sa*FabI complex: (i) hydrogen bonds between both nitrogens of the pyridoazepinaminium ring and Ala95, (ii) hydrogen bond between the acrylamide linker carbonyl and the active site tyrosine (Tyr156), and (iii) hydrophobic interactions between the benzofuran ring and Tyr146, Pro191, Ile153, Met206 and Phe203.

###### Natural Compounds

**Figure 39:**
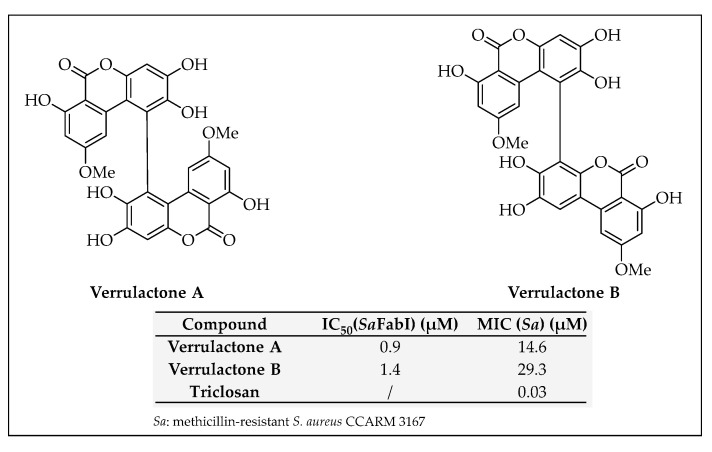
Structure and in vitro activities of **verrulactones A** and **B** [167,168].

Kim et al. isolated **verrulactones A** and **B** (Figure 39) from culture broth of the fungal strain *Penicillium verruculosum* F375 [167]. These alternariol class compounds were both *Sa*FabI inhibitors with IC_50_ values of 0.92 and 1.41 µM, respectively. Furthermore, they both displayed lower ATB activities against methicillin-resistant *S. aureus* (MIC = 14.6–29.3 µM) than **triclosan** (MIC = 0.03 µM) [168].

**Figure 40:**
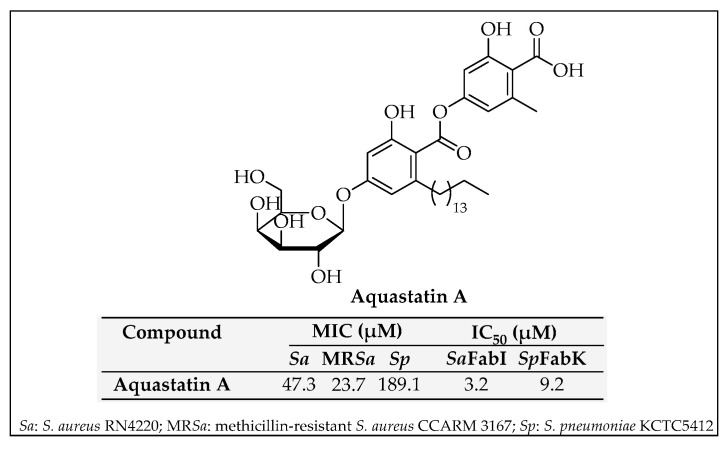
Structure and in vitro activities of **Aquastatin A** [169].

In 2009, Kwon et al. isolated **Aquastatin A** (Figure 40) from the fungus *Sporothrix* sp. FN611 [169]. This compound slightly inhibited the growth of both methicillin-resistant and -sensitive *S. aureus* strains (MIC = 23.7 and 47.3 µM, respectively). It targeted *Sa*FabI and *Sp*FabK with IC_50_ of 9.2 and 3.2 µM, respectively. Furthermore, biochemical study indicated that **Aquastatin A** uses a mixed mechanism of inhibition, since it interacts with *Sa*FabI as a free enzyme and with the *Sa*FabI–NADPH complex.

**Figure 41:**
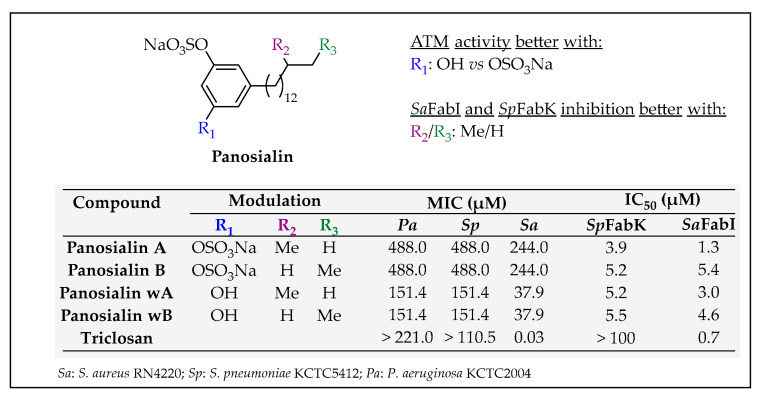
Structure, SAR and in vitro activities of **panosialins A**, **B**, **wA** and **wB** [170].

In 2013, the same team isolated four acyl-benzenediol sulphate metabolites, **panosialins A**, **B**, **wA** and **wB** (Figure 41), from *Streptomyces* sp. AN1761 [170]. These four molecules were active against *Sa*FabI and *Sp*FabK with IC_50_ values in the range of 1.3–5.5 µM. Furthermore, **panosialins A** and **B**, with a sulphate group at R_1_, displayed lower ATM activities than **panosialins wA** and **wB** against *P. aeruginosa*, *S. pneumoniae* and *S. aureus*. In addition, biochemical analysis showed that **panosialin wB** uses a mixed mechanism of enzymatic inhibition.

**Figure 42:**
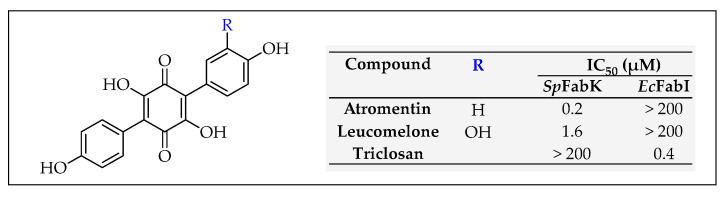
Structures and inhibitory activities of **atromentin** and **leucomelone** [171].

In 2006, the team of Zheng tried to develop *Sp*FabK inhibitors and isolated two terphenyl compounds from the fungus F0110248, isolated from a soil sample that was collected in a corn field around Kongju-city, Chungchongnam-do, Korea: **atromentin** and **leucomelone** (Figure 42) [171]. They both displayed interesting inhibitory activity against *Sp*FabK with IC_50_ of 0.2 and 1.6 µM, respectively. These two compounds showed specific activity against FabK. Unfortunately, they did not exhibit ATM activity against *S. pneumoniae*, *P. aeruginosa* and *E. faecalis*.

#### 2.6. Inhibitors That Target Several FAS-II Enzymes

**Figure 43:**
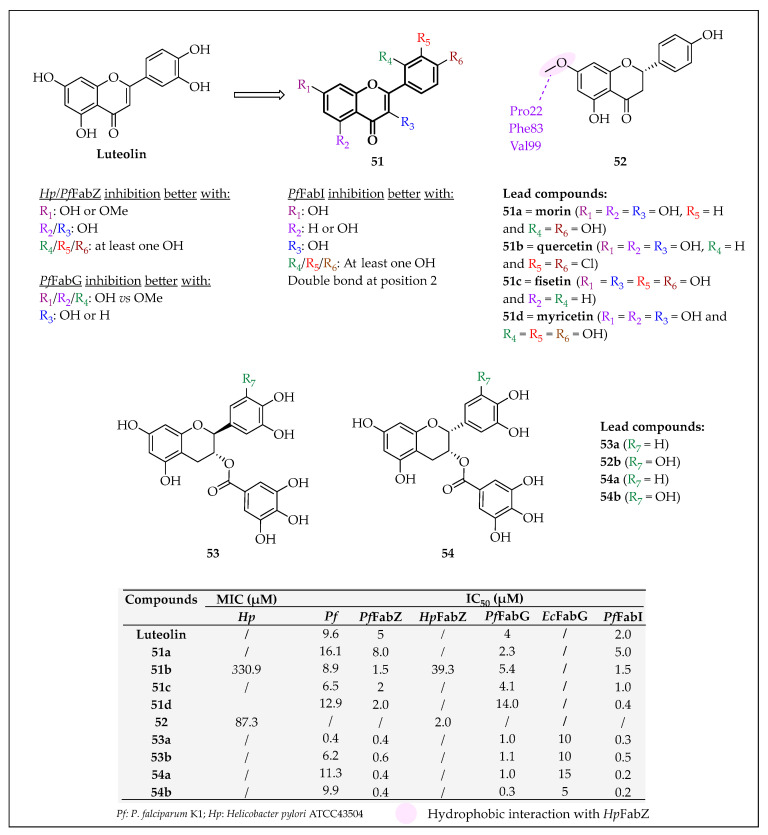
Structure, SAR and representation of main interactions with *Hp*FabZ of flavonoids **48–51** and in vitro activities of lead compounds [103,173,174,176].

Flavonoids, such as polyphenols and catechins, were investigated towards the development of multi-target inhibitors of the FAS-II system enzymes and more precisely FabG, FabI and FabZ. In 2004, Tasdemir et al. identified **luteolin**, a secondary metabolite of the endemic Turkish plant *Phlomis brunneogaleata*, as a *Pf*FabI inhibitor (IC_50_ = 2.0 µM) [172]. This study prompted them to assess a library of twenty-eight polyphenols **51** (Figure 43) as potential *Pf*FabG, *Pf*FabZ and *Pf*FabI inhibitors [173]. Thus, the ATM effects of methylation or H-substitution of the hydroxy groups and modulation of the oxidation states were investigated. When all hydroxy groups were methylated, no activity against *P. falciparum* was observed. The presence of a hydroxy group at the β-position of the ketone did not enhance the activity. Unfortunately, these flavonoids displayed poor antiplasmodial activities (micromolar IC_50_ values against *P. falciparum*). Nonetheless, interesting inhibitory activities were observed, and some SAR were highlighted. Against *Pf*FabG, **51a** (**morin**) was the only flavonoid with better activity than **luteolin** (IC_50_ = 2.3 vs. 4 µM). They demonstrated that, to inhibit *Pf*FabZ, compounds have to possess at least one hydroxy group on the phenyl substituent (R_4_) and another one on the position 5 of the flavone (R_2_). Nonetheless, **52** was 3.8-fold more active against *H. pylori* and ten times more against *Hp*FabZ than **quercetin** (**51b**) [106]. **Quercetin** and **fisetin** (**51c**) were better *Pf*FabZ inhibitors than **52**. A 3-hydroxy substituent (R_3_) should be preferred to inhibit *Pf*FabZ. However, there was no difference in activity between a hydrogen atom or a hydroxy group at the position 5 (R_2_), but no structural data are available for *Pf*FabZ to confirm these experimental observations. Docking studies were performed only for *Hp*FabZ with **52**, and hydrophobic interactions were observed between the methoxy of **52** and three residues of *Hp*FabZ: Pro22, Phe83 and Val99 [174]. This team showed that flavonoids acted as non-competitive inhibitors and that the 7-hydroxy group (R_1_), the double bond at position 2, and at least one hydroxy group on the 2-phenyl ring (R_4_) were necessary to inhibit *Pf*FabI. The lead compound, **51d**, displayed IC_50_ of 0.4 µM against *Pf*FabI and of 12.9 µM against *P. falciparum*.

Among flavonoids, ten catechin gallates **53–54** (Figure 43) were studied to inhibit FabG enzymes [173,175]. The studied catechin gallates displayed weak activity against *P. falciparum*. **54b** was the most potent inhibitor against both *Pf*FabH and *Ec*FabH. Nevertheless, the three other catechin gallates (**53a–b** and **54a**) exhibited IC_50_ values around 1 µM against *Pf*FabG. Additionally, it seemed that the stereochemistry of carbon C2 did not play a significant role in *Pf*FabG inhibition. These four catechins also exhibited interesting *Pf*FabZ inhibitory activity, with IC_50_ values of 0.4–0.8 µM. Furthermore, they were competitive inhibitors of crotonoyl-CoA and very potent *Pf*FabI inhibitors.

## 3. Conclusions

ATM resistance has become a public health issue. Consequently, there is an urgent need for treatments with new modes of action. Fatty acid biosynthesis, particularly the FAS-II system, is a prime target to fight ATM resistance. This system is constituted of eleven potential targets: one transacylase (FabD), three condensing enzymes (FabB, FabF and FabH), one ketoacyl reductase (FabG), two dehydratases (FabA and FabZ) and four enoyl reductases (FabI, FabK, FabL and FabV). Over the last decades, many teams have tried to develop FAS-II enzyme inhibitors. FabI (or InhA in mycobacteria) is the target of two commercial inhibitors: **triclosan** and isoniazid. Despite expanded efforts, only two FAS-II enzyme inhibitors (both of FabI), **afabicin dephosphono** and **CG400549**, are in the clinical pipeline to treat *S. aureus* infections. From **triclosan** and coumarin derivatives to pyridones through imidazoles and some natural compounds such as **Aquastatin A**, FabI is by far the most studied enzyme of the FAS-II system. Nevertheless, for now, FabI inhibitors are selective for bacteria possessing exclusively FabI as ENR, which could be interesting to limit side effects, but it also reduces their spectrum of activity (inactive against *Y. pestis* or *S. pneumoniae*, for instance). However, FabI is not the only enzyme of interest. Indeed, condensing enzymes, mainly FabH, are the target of benzoic acids such as **platencin**, five-membered heterocycles, **TLM** and its derivatives, for instance, or fused cycles which often display broad-spectrum ATM activities. Additionally, the dehydratase FabZ is inhibited by iminophenols and quinolines (**NAS91** family), which exhibit inhibitory activity in a micromolar range (IC_50_). Even if FabG is ubiquitously represented amongst pathogens, only two inhibitors (**CBK261309C** and **CBK066822**) were described, but their ATM activities were not evaluated, probably due to their weak inhibitory activity. Furthermore, some multi-target FAS-II inhibitors were developed using flavonoids, among which the catechins were the most promising. However, no FabA, FabD or FabL inhibitors have been reported yet. Thus, the FAS-II enzymes are definitely interesting and promising targets for future ATM drug development. Many inhibitors display broad-spectrum activities, and some of them are active against strains which are resistant to current ATBs without cytotoxicity. At present, afabicin and its derivative **fabimycin** appear to be the most promising ATM drug candidates.

## Data Availability

Data sharing not applicable.

## Abbreviations

The following abbreviations are used in the review:

ACP	acyl carrier protein
ATB	antibacterial
ATM	antimicrobial
CoA	coenzyme A
ED	electron-donating
ENR	enoyl-ACP reductase
EW	electron-withdrawing
FAS	fatty acid synthase
IC_50_	half inhibitory concentration
INH	isoniazid
KAS	ketoacyl-ACP synthase
MIC	minimal inhibitory concentration
NAC	2-decenoyl-*N*-acetylcysteamine
NADH	nicotinamide adenine dinucleotide
NADPH	nicotinamide adenine dinucleotide phosphate
PDB	Protein Data Bank
SAR	structure–activity relationship
SDR	short-chain dehydrogenase/reductase
TLM	thiolactomycin
WT	wild-type
